# Dawson- and Lindqvist-Type Hybrid Polyoxometalates: Synthesis, Characterization and Ca^2+^-ATPase Inhibition Potential

**DOI:** 10.3390/molecules30224334

**Published:** 2025-11-07

**Authors:** Islem Meskini, Frédéric Capet, Gil Fraqueza, Necmi Dege, Muhammad Nawaz Tahir, Brahim Ayed, Manuel Aureliano

**Affiliations:** 1 Laboratory of Physico-Chemistry of Materials LR01ES19, Faculty of Sciences, University of Monastir, Monastir 5019, Tunisia; 2 Université Lille, CNRS, Centrale Lille, ENSCL, Université Artois, Unité de Catalyse et Chimie du Solide, F-59000 Lille, France; 3 Instituto Superior de Engenharia, Universidade do Algarve, 8005-139 Faro, Portugal; gfraque@ualg.pt; 4 Centro de Ciências do Mar do Algarve (CCMAR/CIMAR LA), Universidade do Algarve, Campus de Gambelas, 8005-139 Faro, Portugal; 5 Department of Physics, Ondokuz Mayıs University, 55200 Samsun, Turkey; 6 Département de Physique, Université de Sargodha, Sargodha 40100, Punjab, Pakistan; 7 Faculdade de Ciências e Tecnologia (FCT), Universidade do Algarve, Campus de Gambelas, 8005-139 Faro, Portugal

**Keywords:** polyoxometalates, Ca^2+^-ATPase inhibitors, POMs synthesis, coupled enzyme pyruvate kinase/lactate dehydrogenase assay, structure-activity correlations

## Abstract

Polyoxometalates (POMs) represent a broad class of anionic inorganic (V, Mo, W) clusters with versatile structures of chemical and physical properties. POMs are inhibitors of many enzymes, including P-type ATPases, well-known to be a target of several approved drugs. Herein, two new hybrid POMs with Mo and mixed V/W, namely (C_2_H_8_N_1_)_6_[V_2_Mo_18_O_62_].3H_2_O (1) and (C_4_H_16_N_3_)_4_[V_2_W_4_O_19_]_3_.12H_2_O (2), were synthesized via wet chemical methods in aqueous solution, and their purity was confirmed and characterized by single X-ray diffraction and infrared spectroscopy. The cations are dimethylammonium ((C_2_H_8_N)^+^) and diethylenetriammonium ((C_4_H_16_N_3_)^3+^), respectively. POMs biological activities were investigated, specifically their inhibitory potential against Ca^2+^-ATPase. The sarcoplasmic reticulum Ca^2+^-ATPase activities were measured spectrophotometrically using the coupled enzyme pyruvate kinase/lactate dehydrogenase assay. For the Ca^2+^-ATPase activity, Dawson (1) showed an IC_50_ value of 3.4 μM, whereas Lindqvist (2) displayed a value of 45.1 μM. The Ca^2+^-ATPase inhibitory potential of these POMs can be correlated with the net charge (namely 6- and 4-) and the charge density (namely 0.33 and 0.67). A structure–activity-relationship was established for a series of 17 POMs Ca^2+^-ATPase inhibitors correlating IC_50_ values and POMs net charge and POMs charge density. The described features make Dawson (1) and Lindqvist (2) attractive POMs in a wide range of chemistry fields as well as in biomedical applications.

## 1. Introduction

**Figure 1:**
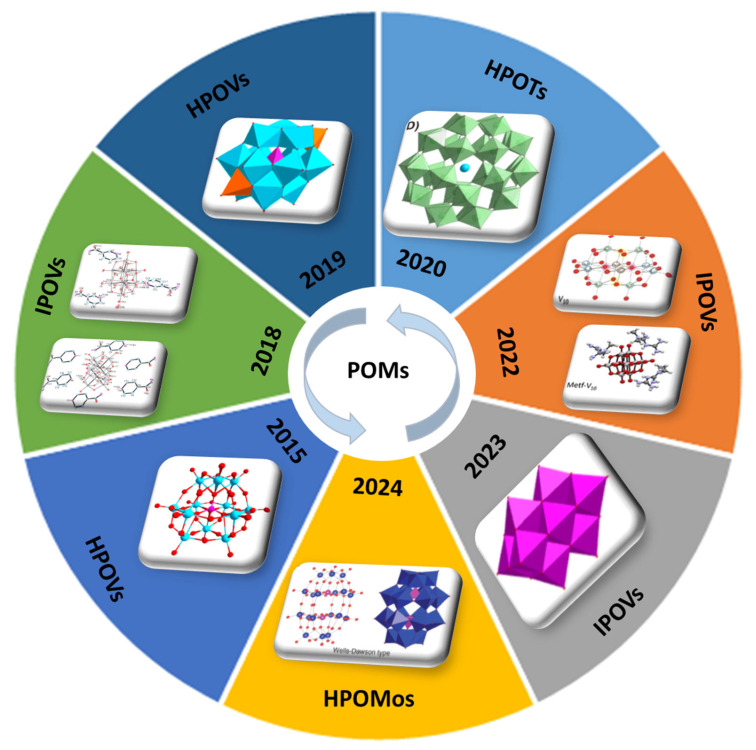
Schematic representation of selected POMs structures reported between 2015 and 2024 [5,6,7,8,9,10,11]; containing Mo, V or W. IPOMs, isopolyoxometalates; HPOMs, heteropolyoxometalates; POVs, polyoxovanadates; POMos, polyoxomolybdates; POTs, polyoxotungstates.

Polyoxometalates (POMs) constitute a large and versatile class of anionic metal-oxide clusters composed of early transition metals, primarily molybdenum (Mo), tungsten (W), or vanadium (V) in their highest oxidation states, interconnected through bridging oxygen atoms. Based on their composition and structure, POMs are broadly categorized into two main types: isopolyoxometalates (IPOMs), which are formed solely from metal-oxo units, and heteropolyoxometalates (HPOMs), which feature one or more heteroatoms (such as phosphorus, silicon, or arsenic) embedded within their frameworks, there by offering greater structural and functional diversity [1,2,3,4] (Figure 1).

**Figure 2:**
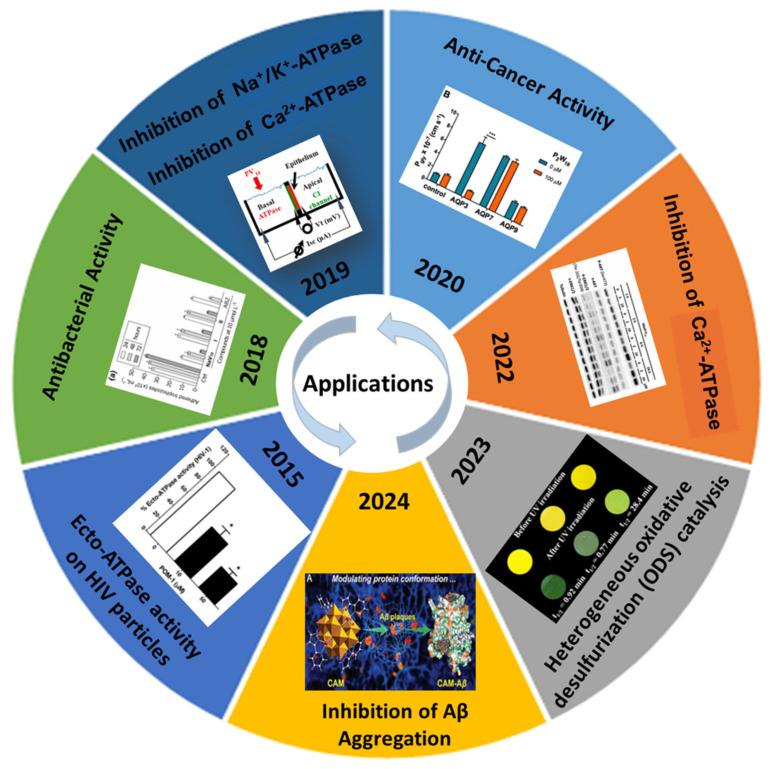
Selected POMs biological and catalytic effects reported between 2015 and 2024, such as ecto-ATPases activity inhibition in HIV particles [5]; antibacterial activity [6]; ex-vivo inhibition of Na^+^/K^+^-ATPase activity [7]; anticancer activity [8]; inhibition of Ca^2+^-ATPase [9]; oxidative desulfurization catalysis [10] and inhibition of ß amyloid (Aß) aggregation [11].

Among the numerous structural motifs, Lindqvist and Dawson architectures have received significant attention due to their well-defined geometries and functional potential in various applications [12,13,14,15,16]. Lindqvist-type POMs, typically with the formula [M_6_O_19_]^n−^ (where M = Mo or W), are small, highly symmetrical clusters composed of six edge-sharing metal-oxo octahedrals. Their condensed structures make them excellent candidates for studies involving molecular recognition, bio-interactions [12], and enzymatic inhibition [13]. Dawson-type POMs, generally expressed as [X_2_M_18_O_62_]^n−^ (X = P, Si, or As), are larger and structurally more complex, consisting of 18 metal-oxo units surrounding two heteroatoms. These features contribute to a greater number of redox-active sites and an expanded surface area, which enhances their utility in catalysis [14] and biological systems [15], particularly where multivalent interactions are beneficial (Figure 2).

The hybridization of POMs with organic moieties via covalent bonding, ionic interactions, or coordination has further broadened their applicability [16]. Moreover, hybridization enables the selective targeting of biomolecules, making these materials promising platforms for biomedical applications, such as enzyme inhibition, drug delivery, and molecular imaging. Beyond biology, hybrid POMs are also being explored for advanced roles in catalysis, materials chemistry, and nanotechnology [17,18,19].

The interaction of POVs with proteins was reviewed in detail [20]. However, recently, several studies described interactions between oxidovanadium complexes and proteins resulting in redox transformations which resulted in the oxidation of V^IV^ to V^V^ and to the formation of a series of POVs that interact and can be formed within the protein structure [21,22]. In fact, the complex chemistry of vanadium is very well known, and that is why the hydrolysis, ligand exchange, redox properties of vanadium compounds and its implications of solution transformation on biological systems, therapeutics, and the environment have been recently revised [23].

The main role of the sarcoplasmic reticulum (SR) Ca^2+^-ATPase is translocation of cellular Ca^2+^ from the cytoplasm to the SR, which is involved in muscle relaxation [24]. However, Ca^2+^-ATPase is globally associated with cellular calcium homeostasis, a process of ion transport that is coupled with ATP hydrolysis. ATP hydrolysis follows a well-known mechanism traversing at least four intermediate steps and two protein conformations, namely E1 and E2, with E1 being the conformation with high affinity for the exported substrate and E2 being the form with high affinity for the imported substrate [24]. As SR vesicles from skeletal muscle contain a large amount of Ca^2+^-ATPase, they represent a useful in vitro model to study the effects of drugs and POMs on calcium homeostasis [25,26]. Moreover, Ca^2+^-ATPases are transmembrane enzymes critical for regulating intracellular calcium levels, and their inhibition is a strategic target for modulating calcium-dependent signaling pathways. In fact, besides targeting these E1E2 ATPases, POMs were also described as an agonist of P2X purinergic receptors from neuron cells, inducing changes in intracellular concentrations through different signaling pathways [27]. In the context of Ca^2+^-ATPase inhibition, both Lindqvist and Dawson-type POMs have shown considerable promise.

Lindqvist-type POMs, owing to their small size and symmetrical configuration, possess favorable diffusion properties that support efficient interaction with enzyme surfaces, facilitating mechanistic studies of enzyme inhibition [13]. In contrast, Dawson-type POMs offer larger and more interactive surfaces, along with multiple redox centers, which enable stronger and potentially multi-site binding to enzymatic active or regulatory regions [13]. These structural distinctions not only affect the binding affinity and inhibitory strength of each type, but also provide complementary insights into the modulation of Ca^2+^-ATPase activity. Such comparative studies are essential for advancing the rational design of POM-based inhibitors with therapeutic potential in calcium-related disorders. Herein, we analyzed for the first time the effects of two hybrid polyoxotungstates (POTs) at the sarcoplasmic reticulum (SR) Ca^2+^-ATPase activity. Specifically, this study aims to (i) synthesize and characterize two novel hybrid polyoxometalates belonging to the Dawson and Lindqvist structural families, namely (C_2_H_8_N)_6_[V_2_Mo_18_O_62_]·3H_2_O (compound 1) and (C_4_H_16_N_3_)_4_[V_2_W_4_O_19_]_3_.12H_2_O (compound 2), respectively; (ii) identify which specific hybrid POT is a more potent inhibitor and (iii) explore structure-activity correlations for all the POMs described in the literature using the same experimental model.

## 2. Results and Discussion

### 2.1. Synthesis

**Figure 3:**
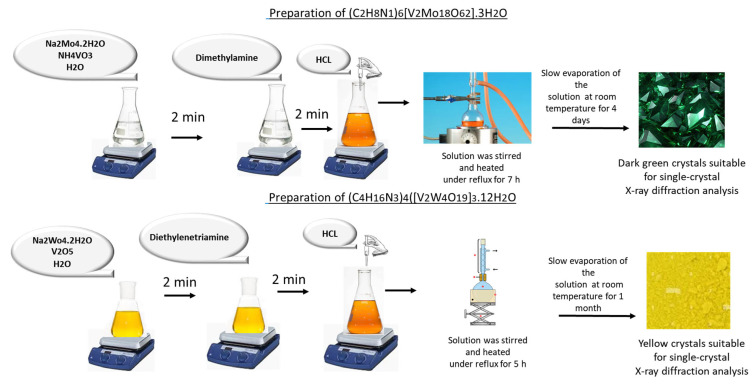
Schematic representation of the synthesis of POMs: (C_2_H_8_N)_6_[V_2_Mo_18_O_62_]·3H_2_O (1) and (C_4_H_16_N_3_)_4_[V_2_W_4_O_19_]_3_.12H_2_O (2).

Compound 1 was synthesized by dissolving sodium molybdate dihydrate (Na_2_MoO_4_·2H_2_O, ≥99%, Sigma-Aldrich, Darmstadt, Germany) (0.65 g, 2.69 mmol) and ammonium metavanadate (NH_4_VO_3_, ≥99%, Alfa Aesar, Ward Hill, MA, USA) (0.1 g, 0.86 mmol) in 30 mL of distilled water. After stirring for 2 min, dimethylamine ((CH_3_)_2_NH, 40 wt.% in water, Sigma-Aldrich, Darmstadt, Germany) (0.65 mL, 9.8 mmol) was added to the solution, and the pH was adjusted to 4 using hydrochloric acid (HCl, 37%, Merck, Darmstadt, Germany). After HCl addition the solution changes color to orange (Figure 3). The mixture was stirred and refluxed for 7 h. Slow evaporation of the solution at room temperature over 4 days yielded dark green crystals suitable for single-crystal X-ray diffraction analysis. (yield: 0.35 g, 42%; yield based on Mo: 95%; yield based on V: 33%).

Compound 2 was synthesized under conditions similar to those of compound 1 (Figure 3). Sodium tungstate monohydrate (Na_2_WO_4_·H_2_O, ≥99%, Sigma-Aldrich, Darmstadt, Germany) (1.32 g, 4.23 mmol) and vanadium(V) oxide (V_2_O_5_, 99.6%, Alfa Aesar, Ward Hill, MA, USA) (0.10 g, 0.55 mmol) were dissolved in 30 mL of distilled water. The pH of the solution was adjusted to 4 by the dropwise addition of hydrochloric acid (HCl, 37%, Merck, Darmstadt, Germany), followed by the addition of diethylenetriamine (H_2_NCH_2_CH_2_NHCH_2_CH_2_NH_2_, ≥99%, Sigma-Aldrich, Darmstadt, Germany) (0.65 mL, 6.17 mmol) instead of dimethylamine. The reaction mixture was stirred under reflux for 5 h. The resulting solution was left to evaporate slowly at room temperature for one month, yielding yellow crystals suitable for single-crystal X-ray diffraction analysis. (yield: 0.50 g, 38%; yield based on W: 36%; yield based on V: 69%).

### 2.2. Crystallization of the Compounds

Single crystals of the compounds were obtained by slow evaporation of an aqueous solution of the purified product at room temperature. The reaction mixture was filtered to remove any insoluble impurities, and the filtrate was left undisturbed for several days until well-formed crystals suitable for single-crystal X-ray diffraction analysis were obtained. The crystals were then carefully collected and dried under ambient conditions. Single-crystal X-ray diffraction data were collected at 109 K for compound 1 using a Bruker PHOTON III DUO diffractometer (Bruker AXS GmbH, Karlsruhe, Germany) equipped with a micro-focus sealed X-ray tube, and at 296 K for compound 2 using a Bruker Kappa APEXII CCD diffractometer (Bruker AXS GmbH, Karlsruhe, Germany) equipped with a fine-focus sealed X-ray tube.

### 2.3. Characterization of the Compounds

**Figure 4:**
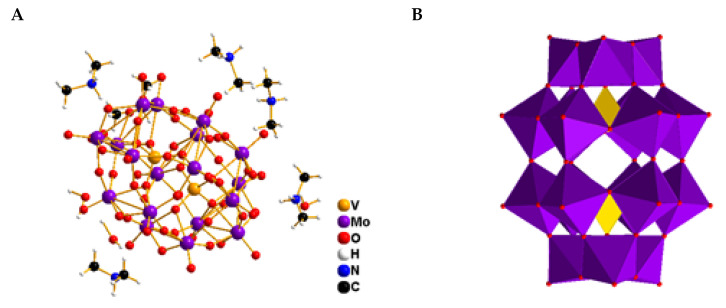
Views of the asymmetric unit contents for the crystal structures of compounds (1) (**A**), and its polyhedral representation (**B**). Color code for B: red, oxygen; purple, Mo; yellow, V.

The crystal structure of compound 1 reveals that it crystallizes in the monoclinic space group P2_1_/n. This novel hybrid material incorporates a Dawson-type polyoxometalate, [V_2_Mo_18_O_62_]^6−^, charge balanced by six dimethylammonium cations (C_2_H_8_N^+^) and accompanied by three lattice water molecules (Figure 4A).

The adjacent layers are additionally stabilized through an extensive network of hydrogen bonds involving the protonated dimethylammonium (C_2_H_8_N^+^) cations, leading to the formation of a three-dimensional open-framework architecture. It is created by close packing of oxygen atoms with V and Mo atoms in the distorted tetrahedral and octahedral voids, respectively. In the Dawson-type polyoxometalate (POM) [28], the molybdenum atoms exhibit two distinct structural environments: six ‘cap’ Mo atoms are located on vertical mirror planes, arranged in two groups of three, while the remaining twelve equatorial Mo atoms are organized into two sets of six but are not situated on any mirror plane [28] (Figure 4B). The cluster contains four distinct types of oxygen atoms based on their coordination modes: (i) eighteen terminal oxygen atoms each coordinate to a single Mo atom, with Mo–O bond lengths ranging from 1.675(5) to 1.795(5) Å; (ii) thirty-six bridging oxygen atoms are each shared between two Mo atoms, exhibiting Mo–O distances between 1.803(5) and 1.994(5) Å; (iii) six triply bridging oxygen atoms are coordinated to one V atom and two Mo atoms, with Mo–O bond lengths ranging from 2.290(12) to 2.377(13) Å; (iv) finally, two μ_4_-oxygen atoms bridge one V atom and three Mo atoms, with Mo–O distances varying between 1.7551(4) and 2.306(4) Å. The variations in the O–Mo–O bond angles, deviating from the ideal values of 90° for cis-oriented oxygen atoms and 180° for trans-oriented oxygen atoms, indicate the degree of distortion within the MoO_6_ octahedra. Specifically, cis O–Mo–O angles range from 71.31(16)° to 135.031(2)°, while trans angles span 148.8(3)° to 170.0(2)° (Table S1).

All molybdenum centers exhibit octahedral coordination geometries, albeit with varying degrees of distortion. The Mo atoms are shifted into the direction of the terminal oxygen atoms away from the centers of the octahedra. The bond length and bond-angle distortion indices were calculated following the Baur method [29]. The calculated distortion indices fall within the ranges of 0.055–0.113 for ID (Mo–O) and 0.223–0.229 for ID (O–Mo–O), reflecting moderate distortions in the coordination environment of molybdenum. The Mo···Mo separations between corner-sharing MoO_6_ octahedra range from 3.668(1) to 3.866(4) Å, which are significantly longer than those observed for edge-sharing octahedra [Mo···Mo = 3.310(4)–3.387(4) Å]. Overall, the Mo–O bond lengths and Mo···Mo distances observed within the polyoxomolybdate framework of the hybrid compound are consistent with previously reported data for Dawson-type structures [30]. The Mo-V distances are in the 3.5262(12)–3.5827(12) Å range. The V-O bond lengths and O-V-O bond angles differ only slightly in the compounds and are in good agreement with those bond lengths and angles found in other compounds containing the (P_2_Mo_18_O_62_)^6−^ anion [31]. Crystallographic data and refinement details are summarized in Table S5.

**Figure 5:**
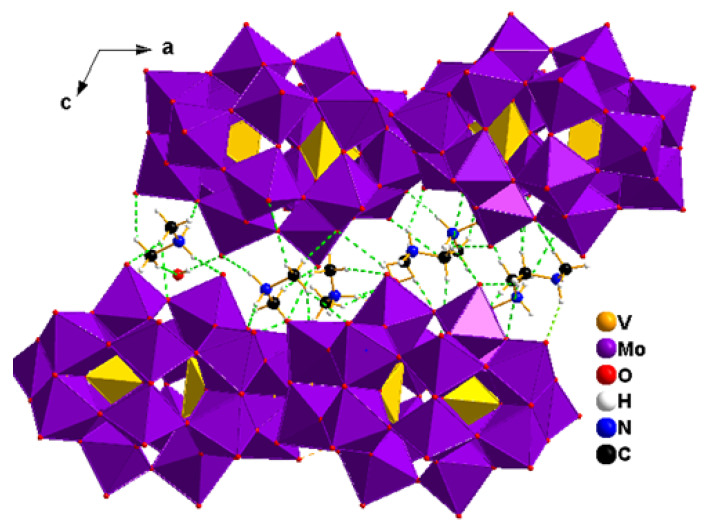
Hydrogen bonding interactions between the organic cations, water molecules, and terminal oxygen atoms of the polyanions in compound (1) along the [010] direction. Green dashed lines represent hydrogen bonds.

Bond valence sum (BVS) calculations performed using the Brown and Altermatt approach [32] indicate that all tungsten centers exhibit valence sums ranging from 5.969 to 6.422, with an average value of 6.045, closely matching the expected oxidation state of +6 for W^6+^. The calculated bond valence sums for the V^5+^ atoms are 5.134 and 4.986, respectively. Within the polyanion, terminal oxygen atoms display bond valences in the range of 1.690–1.823 valence units, while bridging oxygen atoms exhibit values between 1.651 and 2.030 valence units. The [V_2_Mo_18_O_62_]^6−^ anions, together with dimethylammonium counterions and lattice water molecules, engage in extensive hydrogen-bonding interactions (Table S3). These interactions involve N–H···O, O–H···O, and C–H···O contacts, giving rise to one-dimensional hydrogen-bonded chains oriented along the crystallographic [010] direction (Figure 5). The measured bond lengths and angles (Table S1) are consistent with values reported for analogous systems containing dimethylammonium cations and water molecules [33].

**Figure 6:**
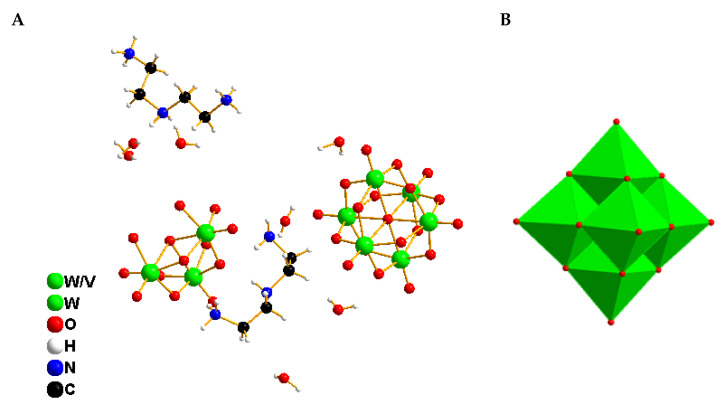
Views of the asymmetric unit contents for the crystal structures of compound 2 (**A**), and its polyhedral representation (**B**).

In contrast, compound 2 crystallizes in the monoclinic space group *P*2_1_/*c* and features discrete Lindqvist-type [V_2_W_4_O_19_]^4−^ anions. Charge neutrality is achieved by the incorporation of protonated diethylenetriamine cations (C_4_H_16_N_3_)^3+^. The crystal structure also contains six co-crystallized water molecules and one neutral diethylenetriamine molecule. The geometric parameters of the structure (Table S2) closely match those previously reported for systems involving the diethylenetriamine cation [34] (Figure 6A).

The [V_2_W_4_O_19_]^4−^ anion retains its canonical highly symmetric structure, composed of six edge-sharing MO_6_ octahedra centered around a μ_6_-oxo atom. The M–O bond distances reveal that the shortest ones correspond to the metal–terminal oxygen bonds, with values ranging from 1.638(12) to 1.711(11) Å. In contrast, the M–Oc distances, involving central oxygen atoms, are the longest, falling between 2.282(11) and 3.2335(15) Å. The M–Ob distances, involving bridging oxygen atoms, exhibit average values between 1.881(11) and 1.975(11) Å (Table S2). The O–M–O bond angles range from 76.1(4)° to 179.7(4)° (Table S2). These structural parameters are consistent with those previously reported for similar Lindqvist-type clusters [35]. To further assess the local environment around the metal centers, the octahedral distortion index (ID) was calculated, yielding values between 0.017 and 0.026 for the W–O_6_ and V–O_6_ octahedra, respectively. These values indicate slight deviations from ideal octahedral geometry, suggesting minor structural relaxation within the polyoxometalate core. Regarding the organic base, precise geometrical parameters were also determined: C–C bond lengths range from 1.45(2)–1.52(2) Å, while C–N bond lengths fall within 1.46(2)–1.53(2) Å. The internal bond angles within the organic fragment vary between 88(10)° and 13(10)°. These values confirm the structural compatibility between the organic cation and the inorganic anion, thereby supporting the stability of the resulting hybrid framework.

**Figure 7:**
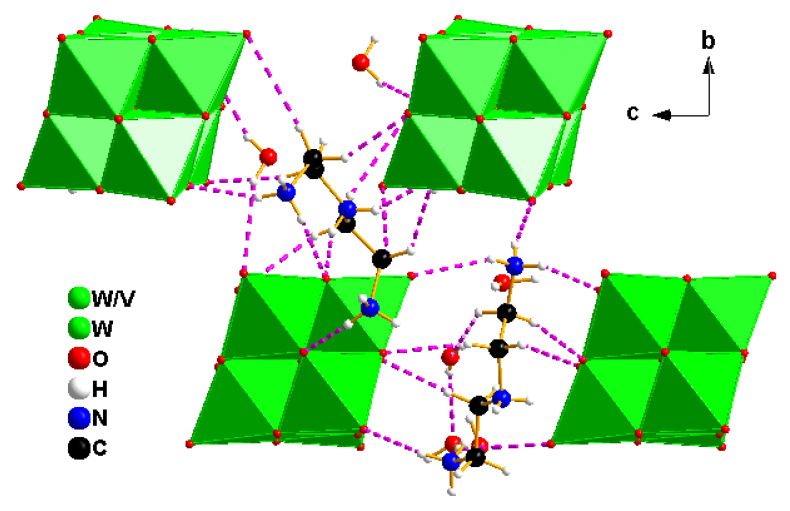
Hydrogen bonding interactions between the organic cations, water molecules, and terminal oxygen atoms of the polyanions in compound (2) along the [100] direction. Magenta dashed lines represent hydrogen bonds.

The [V_2_W_4_O_19_]^2−^ anions are involved in a network of non-covalent interactions, primarily governed by electrostatic forces with the organic counterions. Additionally, terminal and bridging oxygen atoms of the POM core participate in hydrogen bonding interactions with protonated amine groups (N–H···O, C–H···O) and co-crystallized water molecules O–H···O (Table S4). These interactions contribute to the formation of a layered supramolecular architecture, where alternating layers of polyoxometalate anions and organic cations stack along the *a*-axis, resulting in infinite one-dimensional chains (Figure 7).

**Figure 8:**
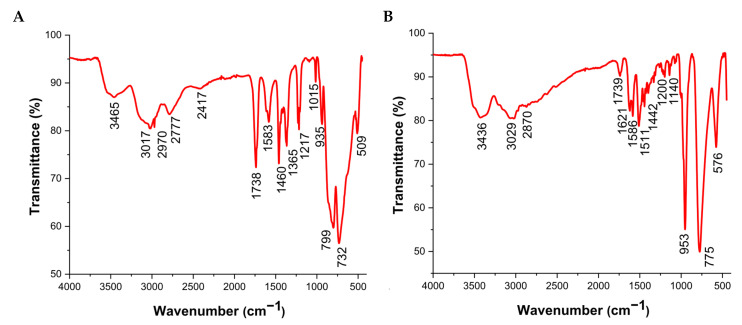
The experimental infrared spectra of compounds 1 (**A**) and 2 (**B**), respectively, (C_2_H_8_N_1_)_6_[V_2_Mo_18_O_62_].3H_2_O and (C_4_H_16_N_3_)_4_[V_2_W_4_O_19_]_3_.12H_2_O.

The IR spectrum for both title compounds (Figure 8) exhibits characteristic vibrational bands, allowing for the assignment of specific functional groups within these hybrid materials. For Dawson-type hybrid polyoxometalate, a band at 935 cm^−1^ is attributed to a combination of ν(M–Ot) stretching vibrations. For Lindqvist-type, this combination appears in the 1140–953 cm^−1^ range. The asymmetric stretching vibration, νas(M–Ot), is observed at 732–799 cm^−1^ for 1, while in the Lindqvist-type compound it appears at 775 cm^−1^. Furthermore, the symmetric stretching vibration, νs(M–Ot), is found at 509 cm^−1^ for Dawson, and at 576 cm^−1^ for 2 [36].

Detailed assignments are described at Table 1. The presence of water molecules is confirmed by the ν(O—H) stretching vibrations observed at 3465 cm^−1^ for the Dawson-type material and 3436 cm^−1^ for compound 2. The corresponding δ(O—H) bending modes are detected at approximately 1583 cm^−1^ for 1 and 1621–1586 cm^−1^ for Lindqvist, consistent with data from the literature [37]. Additional peaks between 1000–3029 cm^−1^ are attributed to the various vibrational modes of the organic cations — the dimethylammonium (C_2_H_8_N_1_)_6_^+^ in compound 1 [38] and the diethylenetriamine (C_4_H_16_N_3_)^3+^ cation in the Lindqvist-type structure [39]. The observed frequencies and their relative intensities for all bands show good correlation with values from the literature for analogous compounds, further supporting the structural data obtained from single-crystal X-ray diffraction (SC-XRD).

### 2.4. Polyoxometalates Inhibition of Ca^2+^-ATPase

In order to evaluate the inhibition potential for each of the POMs compounds regarding the Ca^2+^-ATPase activity, increasing concentrations of each POM and a control without the presence of the inhibitor were used in order to determine the IC_50_ values. For each of these assays, an absorbance versus time kinetic is obtained. In the absence of the POM, the control assay was obtained—that is, a kinetic with a higher slope, while by increasing the concentrations of the POMs, lower enzymatic kinetic slopes were found due to the inhibition of the ATPase activity (Figure S1).

**Figure 9:**
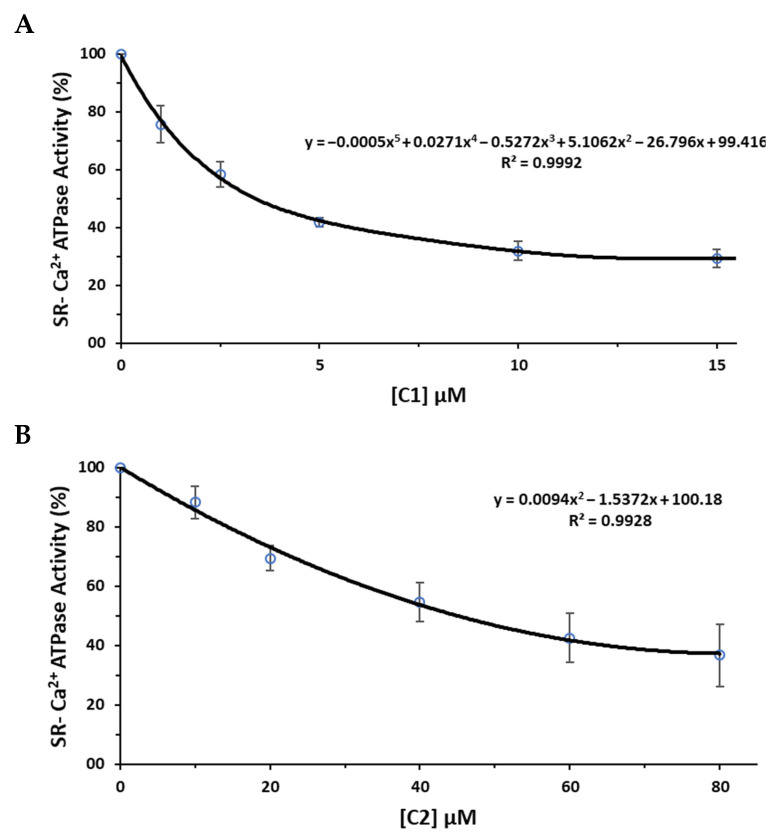
Inhibition curve of SR-Ca^2+^-ATPase activity used for the determination of the IC_50_ values of compounds 1 (**A**) and 2 (**B**). Three independent assays were performed for each concentration. IC_50_ value corresponds to inhibitor concentration needed for obtaining 50% of inhibition of control in the absence of the inhibitor.

Using this method, the effects of the inhibitors can be observed in real time and recorded within 2 to 5 min upon addition of the inhibitor, as described previously [13,25,26]. With these data, the SR Ca^2+^-ATPase activity percentage and a graph for Ca^2+^-ATPase in percentage versus concentration of the inhibitor, POMs compounds, can be obtained (Figure 9). For each compound, the equation governing the trendline of this can be solved for 50% in order to determine the IC_50_ value. The results obtained for compound (1) showed an IC_50_ value of 3.4 μM, whereas compound (2) displayed a value of 45.1 μM (Figure 9).

SR Ca^2+^-ATPase vesicles are a well-known model to study the effects of clinically approved drugs on the activity of Ca^2+^-ATPase and consequently on the effects on calcium translocations and homeostasis with implications in several disease treatments [40,41,42]. Moreover, Ca^2+^-ATPase was described as a putative POM target in cancer [15], among other enzymes associated with several physiological and pathological processes.

Ca^2+^ ions are the major intracellular second messenger involved in a multitude of cellular functions [43]. A growing number of studies identify dysregulation of calcium (Ca^2+^) homeostasis, particularly the anomalous increase in cytosolic Ca^2+^ concentrations, as being implicated in the cascade of pathophysiological events that trigger the clinical picture for Alzheimer’s disease, for example [44]. Thus, the regulation of intracellular Ca^2+^ levels is tightly controlled by an intricate variety of channels, pumps, exchangers and buffering systems, which act in a coordinated manner to maintain the balance necessary for optimal neuronal performance. This state of balance gives rise to name of calcium homeostasis [45].

One of the components of these regulatory mechanisms is Ca^2+^-ATPases, a family of transport proteins that meticulously calibrate calcium levels within the cell [46]. These molecular pumps work incessantly, promoting the extrusion of calcium from the cytosol by active transport, either through the plasma membrane to the extracellular environment by plasma membrane Ca^2+^-ATPases (PMCA), or for intra-cellular storage in the endoplasmic reticulum (ER) by the action of sarcoplasmic/endoplasmic reticulum Ca^2+^-ATPases (SERCA), or in the Golgi complex mediated by secretory pathway Ca^2+^-ATPases (SPCA) [44]. Driven by ATP hydrolysis, these pumps contribute to the reestablishment of resting Ca^2+^ concentrations after the action potential, and to the maintenance of calcium homeostasis [46].

Disruption of these pumps or due to pathological influx of Ca^2+^, induces a state of chronic elevation of intracellular calcium concentration [47,48]. The consequences of imbalanced calcium homeostasis are profound, precipitating a cascade of deleterious events that include the activation of enzymatic pathways that lead to cell death, the production of ROS and the induction of inflammatory responses, accelerating the progression of, for example, AD [43]. Recently, polyoxometalates (POMs) were described to present agonistic properties on purinergic P2 receptors from neuron cells [27]. Thus, POMs inhibited the P-type ATPases [13,26] and also modulated the cytosolic calcium concentrations in neurons, pointing out a useful tool in the studies of pathological processes of AD [25,27,41,42,43,44,45,46,47,48].

Besides POMs, thapsigargin (TG), celecoxib (CE), and cyclopiazonic acid (CPA) are well-known clinically approved drugs that target P-type ATPases such as the Ca^2+^-ATPase. In fact, for Ca^2+^-ATPase, TG showed an IC_50_ of 0.001–0.029 µM, CPA an IC_50_ of 0.1–0.2 µM, and celecoxib an IC_50_ of 35 μM. Besides the organic compounds, Ca^2+^-ATPase vesicles were used to study the inhibition potential of inorganic compounds. Thus, several inorganic compounds were described as potent Ca^2+^-ATPases inhibitors, such as POMs and metals complexes with IC_50_ values between 0.3 to 200 μM, which are comparable with the ones described in the present paper [7,9,13,25,26,40]. Table 2 summarizes some of the IC_50_ values found for 17 POMs regarding Ca^2+^-ATPase activity, determined with the same experimental model [7,9,13,25,26,49].

**Figure 10:**
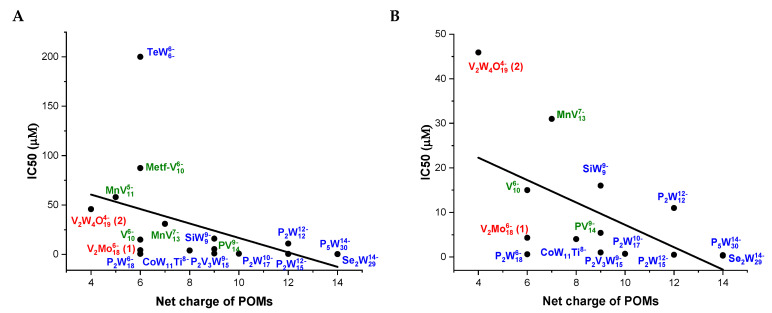

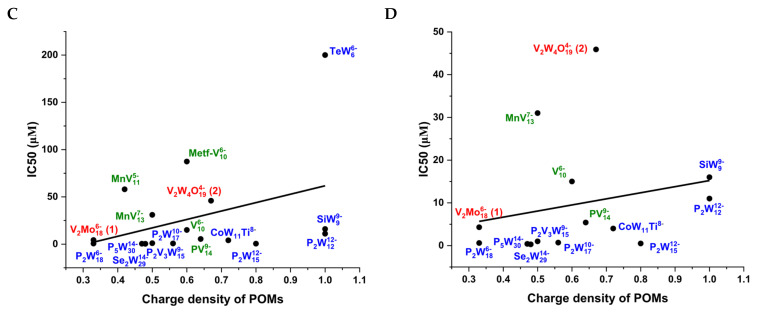
Structure–activity correlations of the several POMs for Ca^2+^-ATPase inhibition. (**A**) Correlation between the Ca^2+^-ATPase IC_50_ values and POMs net charge; (**B**) Correlation between the Ca^2+^-ATPase IC_50_ values lower than 50 μM of inhibition and their net charge; (**C**) Correlation between the Ca^2+^-ATPase IC_50_ values and their charge density, expressed as charge of the POM divided by its number of metal addenda atoms; (**D**) Correlation between the IC50 values lower than 50 μM of inhibition and their charge density, expressed as charge of the POM divided by its number of metal addenda atoms. In red we showed the position for compounds 1 and 2, in blue the POTs and in green the POVs.

As can be observed in Table 2 compound 1, V_2_Mo_18_ (Wells-Dawson), presents an IC_50_ value of inhibition (3.4) higher than other POTs Wells-Dawson type of structures, such as P_2_W_18_, Se_2_W_29_ and P_2_V_3_W_15_ (all below 1 µM). On the other hand, compound 2 (Lindqvist) shows to be four times more potent that the Anderson type (TeW_6_). However, it has a similar inhibition potential than other structure types of POMs, such as the MnV_11_ (58 µM) Keggin type. Structure–activity correlations between the IC_50_ values of inhibition and their charge and charge density expressed as charge of the POM divided by its number of metal addenda atoms can be found in Figure 10. The charge densities for POMs clusters were calculated as the ratio between the POM charge q and the number of tungsten/vanadium/molybdenum atoms *m* as referred to in Table 2. The lower Ca^2+^-ATPase inhibitory potential for V_2_W_4_ in comparison to V_2_Mo_18_, which is 45.1 and 3.4 μM, respectively, can be eventually explained because it presents a lower net charge and a higher charge density (Figure 10). In fact, it can be observed that increasing the POMs negative net charge from −4 to −14 favors the Ca^2+^-ATPase inhibition potential of the POMs. The IC_50_ values are lowest for the POMs with higher negative charges, such as Se_2_W_29_ and P_5_W_30_, although IC_50_ values below 1 μM can also be found for POMs with net charge of 6 — such as for P_2_W_18_ and P_2_V_3_W_15_ (Figure 10A,B).

Conversely, the different ATPase inhibition potential of V_2_W_4_ and V_2_Mo_18_ can also be explained by the POMs charge density. By increasing the charge density, it was observed that the ATPase inhibition potential decreases (Figure 10C,D). V_2_Mo_18_^6−^ shows an q/*m* value of 0.33, the same as for P_2_W_18_ and P_2_V_3_W_15_ (Table 2). In fact, POMs with moderate charge density (q/*m*= 0.33) have been suggested to interact strongly with various surfaces of different or mixed polarities, as presented by a lipid bilayer and/or a protein molecule favoring a chaotropic effect [50]. Still, both the Preyssler-type anion and Se_2_W_29_^14−^ with a similar charge density (q/*m* = 0.47 and 0.48, respectively) show the highest inhibitory potential, at IC_50_ = 0.37 and 0.3 μM, respectively. On the other hand, the two Wells-Dawson anions V_2_Mo_18_^6−^ and W_15_V_3_^6−^ have the same q/*m* value of 0.33, but their inhibition potentials are slightly different, probably due to the presence of substituting V ions, which changes the POMs charge distribution and possibly the binding mode as well.

Additionally, the reason why POMs showed stronger inhibitory effect on Ca^2+^-ATPase activity at the molecular level might be due to specific protein interactions, as previously described for the interaction of decaniobate and decavanadate with actin [51]. In this study, it was described that V_10_ binds to actin binding site alfa, the same binding site for the native ligand ATP, probably because Lys/Arg interactions are favored at the binding site, contrary to Nb_10_ presenting a lower charge density [51]. Although strong POMs interactions do not equal strong inhibition, it is plausible that electrostatic interactions play, at least in part, a potential role, whereas metformin-decavanadate has an IC_50_ ATPase inhibition value six times higher than V_10_ alone [9]. It was also demonstrated that, in the case of medium-size POMs where the charge is more than −12 and number of addenda atoms is not higher than 22, POM antibacterial activity mainly depends on the total net charge [52]. Still, further studies are needed to unravel at molecular level why certain POMs showed a stronger inhibitory effect on Ca^2+^-ATPase as well as on Na^+^/K^+^-ATPase and E-NTPDase activity [53,54]. In fact, as previously observed in others’ studies, inhibitory activities and POMs parameters correlations, for example with the same Ca^2+^- ATPase and also with microorganisms and/or cancer cells, although not well defined, provides new insights into future research directions in the field [13,52,55,56].

POMs present well-known distinct types of structures, as described elsewhere [15]. Their activity against cancer, as agents fighting bacterial infection or their ability as inhibitors of Ca^2+^-ATPases was previously compared and correlated with specific POMs features [13,15,52,55,56]. Moreover, the effects of POMs being polyoxotungstates (POTs), polyoxomolybdates (POMos), polyoxovanadates (POVs) or polyoxopaladates (POPds) in cancer cell viability were recently analyzed [57]. When comparing and sorting the IC_50_ values in ascending order, POVs were obtained first, then POTs, POPds, and finally, POMos [57]. Among the POMs studied in biological systems, the POV decavanadate (V_10_), an isopolyoxovanate (Figure 1), is perhaps the most studied [20]. However, another POM that should be mentioned is the Preyssler type P_5_W_30_ (Figure 1). Besides the several biological activities summarized elsewhere [8], P_5_W_30_ was recently described to act as an agonist of purinergic P2 receptors in neurons [27]. Regarding POMs effects in neurodegenerative diseases, for example, Alzheimer’s disease (AD), the most referred to in the AD studies were the Keggin types W_12_ and W_11_, as well as the Wells-Dawson type W_18_ [58,59,60]. Also recently, a mixed-valence (MV) polyoxovanadates (V_15_) that can adopt various structural patterns, including wheel and bowl-type structures, was described to present several biological activities, such as Ca^2+^-ATPase ability and anti-breast cancer activity, besides the ones described elsewhere [55].

By combining POMs with other organic components, hybrid POMs might enhance biological activity, improve biocompatibility and reduce toxicity to healthy cells, showing great promise in clinical treatments for cancer, bacterial infections, viral diseases, as well as neurological diseases [9,11,12,15,20,55,56,57,58,59,60]. To our knowledge so far, studies on the use of pure POMs, hybrid POMs, nanoparticles containing POMs, or MOF containing POMs as clinically approved drugs are rare or even absent. However, POMs were recently described in the treatment of skin diseases in humans [61].

In summary, two new hybrid polyoxometalates, Dawson compound 1 and Lindqvist compound 2, were successfully synthesized and characterized using single-crystal X-ray diffraction and infrared spectroscopy. The POMs were analyzed regarding the inhibitory potential for Ca^2+^ATPase activity using a coupled enzymatic method. IC_50_ values of inhibition for POMs concerning a major protein involved in several biological processes and associated with several diseases, the Ca^2+^-ATPase, were obtained. It was determined that compound 1 presented an IC_50_ value of 3.4 μM, that is, about 13-fold inhibition potential regarding ATPase activity than compound 2.

By analyzing Ca2+-ATPase inhibitory activity and the structure of POMs using the same enzymatic assay at the same experimental conditions, a structure–activity-relationship can be established for a series of 17 POMs inhibitors of this ATPase regarding POMs net charge and charge density. Further studies are needed to unravel at a molecular level POMs features that correlate with biological activities.

Using the Ca^2+^-ATPase vesicles model to look for new Ca^2+^-ATPase inhibitors, we aim to push forward future research in the field for other POMs, such as purely inorganic POMs, hybrids with organic components, and also as nanoparticles and/or metal organic frameworks (MOFs) as potential drugs that target ATPases. We therefore aim to advance putative applications in several areas of research.

## 3. Materials and Methods

### 3.1. Reagents

Sodium molybdate dihydrate, Na_2_MoO_4_·2H_2_O (Sigma-Aldrich, Darmstadt, Germany), sodium tungstate dihydrate, Na_2_WO_4_·2H_2_O (Sigma-Aldrich, Darmstadt, Germany), ammonium metavanadate NH_4_VO_3_, (Alfa Aesar, Ward Hill, MA, USA), vanadium oxide V_2_O_5_ (Alfa Aesar, Ward Hill, MA, USA), dimethylamine (Sigma-Aldrich, Darmstadt, Germany), diethylenetriamine Sigma-Aldrich, Darmstadt, Germany), and hydrochloric acid (Merck, Darmstadt, Germany), as well as all other solvents and chemicals obtained from commercial sources, were used as received without any further purification.

### 3.2. Characterization

Single-crystal X-ray diffraction data were collected at 109 K for compound 1 using a Bruker PHOTON III DUO diffractometer equipped with a micro focus sealed X-ray tube, and at 296 K for compound 2 using a Bruker Kappa APEXII CCD diffractometer equipped with a fine-focus sealed X-ray tube. Preliminary diffraction images indicated monoclinic symmetry for both compounds. Systematically absent reflections suggested the space group P2_1_/n for compound 1 and P2_1_/c for compound 2.

The crystal structures were solved by direct methods using the SHELXS-2019 [62] program, which enabled the location of both inorganic and organic atoms. The remaining non hydrogen atoms were located via successive difference Fourier maps using the SHELXL-2019 [63] program. In the final least-squares refinement of atomic parameters, hydrogen atoms were treated using isotropic thermal parameters. The final R-values Were R_int_ = 0.027 for compound 1 and R_int_ = 0.129 for compound 2. Crystallographic data and refinement details are summarized in Table S5.

For further information, the crystallographic data for the synthesized compounds have been deposited with the Cambridge Crystallographic Data Centre (CCDC) under the deposition numbers CCDC 2496911 for compound 1 and CCDC 2497371 for compound 2. Functional groups were identified by Fourier-transform infrared (FT-IR) spectroscopy at room temperature using a Spectrum BX II PerkinElmer spectrometer in the range of 400–4000 cm^−1^.

### 3.3. Preparation of Sarcoplasmic Reticulum Vesicles and POMs Solutions

The sarcoplasmic reticulum vesicles (SRV) that contain the Ca^2+^-ATPase was previously prepared from rabbit skeletal muscles as described elsewhere [13,25,26]. The vesicles were suspended in 0.1 M KCl, 10 mM HEPES (pH 7.0), diluted 1:1 with 2.0 M sucrose, and frozen in liquid nitrogen for storage at −80 °C. SDS-PAGE analysis revealed that Ca^2+^-ATPase accounted for at least 70% of the total protein, as described elsewhere [26]. Stock solutions of the POMs compounds (1 mM) were freshly prepared by dissolving the solid compound in miliQ water and keeping the solution at room temperature before use.

### 3.4. Determination of Ca^2+^-ATPase IC_50_ Values

For carrying out the determination of Ca^2+^-ATPase activity assays, the following medium was prepared: 25 mM HEPES (pH 7.0), 100 mM KCl, 5 mM MgCl_2_ and 50 µM CaCl_2_.

**Figure 11:**
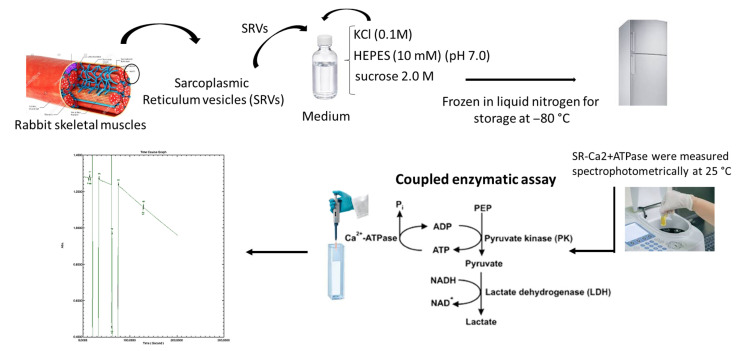
Schematic representation of the procedure used at the study of the effects of POMs on ATP hydrolysis by the Ca^2+^-ATPase. The POMs were added after addition of the medium and before the addition of the enzymes of the coupled assay.

The experiments were performed with isolated sarcoplasmic reticulum vesicles (SRVs) previously diluted to 1 mg/mL with a sucrose concentration of 0.25 M (Figure 11). 0.8 mL of medium described above was added into a quartz cuvette. 0.42 mM phosphoenolpyruvate (PEP), 18 IU lactate dehydrogenase, 7.5 IU 155 pyruvate kinase, and 2.5 mM ATP without (control) or with increasing concentrations of POMs were added to this medium. POMs were added after addition of the medium and before the addition of the enzymes of the coupled assay. The absorbance of the solution was measured spectrophotometrically at 340 nm, 25 °C, and an auto zero was performed. Subsequently, 0.25 mM NADH was added, allowing the absorbance value to stabilize to about 1.3 O.D. The assay begins after the addition of 10 μg/mL of Ca^2+^-ATPase (basal activity) and one minute after ionophore A23187 (calcimycin) 4% (*w*/*w*) was added (uncoupled activity), and the kinetics followed for about 2 min in the absence or in the presence of increasing concentrations of POMs, from 0 to 15 μM for compound 1 and from 0 to 80 μM for compound 2. After the addition of all the components, the final volume was 1 mL.

For each experimental condition, the experiments were always performed in triplicate. Therefore, the determination of Ca^2+^-ATPase activity without and with the inhibitor was made using a continuous enzymatic method using the measurement of absorbance versus time (Figure S1), as described elsewhere [13,25,26].

## Figures and Tables

**Table 1:** Characteristic infrared absorption bands (cm^−1^) of compounds (1) and (2).

Assignment	(C_2_H_8_N_1_)_6_[V_2_Mo_18_O_62_].3·H_2_O	(C_4_H_16_N_3_)_4_[V_2_W_4_O_19_]_3_.12H_2_O
ν M—Ot	935	1140–953
ν_as_ M—Ot	732–799	775
ν_s_ M—Ot	509	576
ν O—H	3465	3436
δ N—H	3017–2970	3029
ν C—N	2417	2870
δ O—H	1583	1621–1586
δ N—H	1460–1365	1511–1442
δ N—C δ C—C	1115–1212	1200

**Table 2:** Comparison of the Ca^2+^-ATPase inhibition potential for 17 POMs containing W, V or Mo, using the same experimental mode, method and conditions for measuring Ca^2+^-ATPase activity.

Compounds	Net Charge	Charge Density	POM Archetype	Ca^2+^-ATPase IC50 (μM)	Reference
P2W18	6-	0.33	Wells-Dawson	0.6	[13]
Se2W29	14-	0.48	Wells-Dawson	0.3	[13]
CoW11Ti	8-	0.72	Keggin	4	[13]
P2W12	12-	1.00	Keggin	11	[13]
SiW9	9-	1.00	Keggin	16	[13]
TeW6	6-	1.00	Anderson	200	[13]
PV14	9-	0.64	Keggin	5.4	[49]
Metf-V10	6-	0.60	Decavanadate	87.4	[9]
MnV11	5-	0.42	Keggin	58	[7]
MnV13	7-	0.50	Keggin	31	[7]
V10	6-	0.60	Decavanadate	15	[26]
P2W15	12-	0.80	Wells-Dawson	0.5	[25]
P2W17	10-	0.56	Wells-Dawson	0.7	[25]
P5W30	14-	0.47	Preyssler	0.4	[25]
P2V3W15	9-	0.50	Wells-Dawson	1.0	[25]
V2Mo18 (1)	6-	0.33	Wells-Dawson	3.4	this study
V2W4O19 (2)	4-	0.67	Lindqvist	45.1	this study

## Data Availability

The original contributions presented in this study are included in the article/Supplementary Material. Further inquiries can be directed to the corresponding author(s).

## Supplementary Materials

The following supporting information can be downloaded at: https://www.mdpi.com/article/10.3390/molecules30224334/s1, Figure S1: Example of experimental registrations of the Ca2+-ATPase inhibition by POMs, using the coupled enzymatic assay. Table S1: Selected bond lengths (Å) and angles (°) for (C2H8N)6[V2Mo18O62].3H2O; Table S2: Selected bond lengths (Å) and angles (°) for (C4H16N3)2(C4H16N3)4[V2W4O19]3.12H2O.; Table S3: Hydrogen bonds (in Å) for (C2H8N)6[V2Mo18O62].3H2O; Table S4: Hydrogen bonds (in Å) for (C4H16N3)4[V2W4O19]3.12H2O; Table S5: Crystal data and structure refinement parameters for compounds 1 and 2.

## Abbreviations

AD	Alzheimer’s Disease
BVS	bond valence sum
CE	celecoxib
CPA	cyclopiazonic acid
E-NTPDase	ecto-nucleoside triphosphate diphosphohydrolases
HPOMs	heteropolyoxometalates
IC50	50% inhibitory concentration
ID	distortion index
IPOMs	isopolyoxometalates
MOFs	metal organic frameworks
MV	mixed valence
PEP	phosphoenolpyruvate
POMos	polyoxomolybdates
POTs	polyoxotungstates
POVs	polyoxovanadates
SRVs	sarcoplasmic reticulum vesicles
TG	thapsigargin

## References

[1] Rathee B., Wati M., Sindhu R., Sindhu S. (2022). Review of Some Applications of Polyoxometalates. Orient. J. Chem..

[2] Zarroug R., Moslah W., Srairi-Abid N., Artetxe B., Masip-Sánchez A., López X., Ayed B., Ribeiro N., Correia I., Corte-Real L. (2025). Synthesis, Crystal Structure, Computational and Solution Studies of a New Phosphotetradecavanadate Salt. Assessment of Its Effect on U87 Glioblastoma Cells. J. Inorg. Biochem..

[3] Bouallegui T., Zarroug R., Artetxe B., Ayed B. (2025). Structural Resolution of the New Polyoxometalate Hybrid (C_2_N_4_H_7_O)_4_(NH_4_)[HMo_7_O_24_]·4H_2_O: Textile Dye Decolorization and BSA Binding Properties. J. Mol. Struct..

[4] Bouallegui T., Dege N., Ayed B. (2023). Structural, Physico-Chemical Properties of a Hybrid Material Based on Anderson-Type Polyoxomolybdates. J. Iran. Chem. Soc..

[5] Schachter J., Delgado K.V., Souza V.B., Bou-Habib D.C., Persechini P.M., Fernandes J.R.M. (2015). Inhibition of ecto-ATPase activities impairs HIV-1 infection of macrophages. Immunobiology..

[6] Missina J.M., Gavinho B., Postal K., Santana F.S., Valdameri G., de Souza E.M., Hughes D.L., Ramirez M.I., Soares J.F., Nunes G.G. (2018). Effects of Decavanadate Salts with Organic and Inorganic Cations on Escherichia coli, Giardia intestinalis, and Vero Cells. Inorg. Chem..

[7] Marques-da-Silva D., Fraqueza G., Lagoa R., Vannathan A.A., Mal S.S., Aureliano M. (2019). Polyoxovanadate Inhibition of Escherichia coli Growth Shows a Reverse Correlation with Ca^2+^-ATPase Inhibition. New J. Chem..

[8] Pimpão C., da Silva I.V., Mósca A.F., Pinho J.O., Gaspar M.M., Gumerova N.I., Rompel A., Aureliano M., Soveral G. (2020). The Aquaporin-3-Inhibiting Potential of Polyoxotungstates. Int. J. Mol. Sci..

[9] De Sousa-Coelho A.L., Aureliano M., Fraqueza G., Serrão G., Gonçalves J., Sánchez-Lombardo I., Link W., Ferreira B.I. (2022). Decavanadate and Metformin-Decavanadate Effects in Human Melanoma Cells. J. Inorg. Biochem..

[10] Aghmasheh M., Rezvani M.A., Jafarian V., Ardeshiri H.H. (2023). Synthesis and Characterization of a New Nanocatalyst Based on Keggin-Type Polyoxovanadate/Nickel-Zinc Oxide, PV14/NiZn2O4, as a Potential Material for Deep Oxidative Desulfurization of Fuels. Energy Fuels.

[11] Ma M., Liu Z., Zhao H., Zhang H., Ren J., Qu X. (2024). Polyoxometalates: Metallodrug agents for combating amyloid aggregation. Natl. Sci. Rev..

[12] Yao C.-G., Zhao Z.-J., Tan T., Yan J.-N., Chen Z.-W., Xiong J.-T., Li H.-L., Wei Y.-H., Hu K.-H., Chen J. (2024). Lindqvist-Type Polyoxometalates Act as Anti-Breast Cancer Drugs via Mitophagy-Induced Apoptosis. Curr. Med. Sci..

[13] Gumerova N.I., Krivosudský L., Fraqueza G., Breibeck J., Al Sayed E., Tanuhadi E., Bijelic A., Fuentes Moreno J., Aureliano M., Rompel A. (2018). The P-Type ATPase Inhibiting Potential of Polyoxotungstates. Metallomics.

[14] Mateos P.S., Ruscitti C.B., Casella M.L., Matkovic S.R., Briand L.E. (2023). Phosphotungstic Wells–Dawson Heteropolyacid as a Potential Catalyst in the Transesterification of Waste Cooking Oil. Catalysts.

[15] Bijelic A., Aureliano M., Rompel A. (2019). Polyoxometalates as Potential Next-Generation Metallodrugs in the Combat Against Cancer. Angew. Chem. Int. Ed..

[16] Wang S.-S., Yang G.-Y. (2015). Recent Advances in Polyoxometalate-Catalyzed Reactions. Chem. Rev..

[17] Chen L., Cui H., Jiang F., Kong L., Fei B., Mei X. (2024). Efficient Removal of Methylene Blue Using an Organic–Inorganic Hybrid Polyoxometalate as a Dual-Action Catalyst for Oxidation and Reduction. Catalysts.

[18] Khalilpour H., Shafiee P., Darbandi A., Yusuf M., Mahmoudi S., Moazzami Goudarzi Z., Mirzamohammadi S. (2021). Application of polyoxometalate-based composites for sensor systems: A review. J. Compos. Compd..

[19] Zhu J.-J., Martinez Soria L., Gomez Romero P. (2022). Coherent Integration of Organic Gel Polymer Electrolyte and Ambipolar Polyoxometalate Hybrid Nanocomposite Electrode in a Compact High Performance Supercapacitor. Nanomaterials.

[20] Aureliano M., Gumerova N.I., Sciortino G., Garribba E., McLauchlan C.C., Rompel A., Crans D.C. (2022). Polyoxidovanadates’ interactions with proteins: An overview. Coord. Chem. Rev..

[21] Ferraro G., Vitale L., Sciortino G., Pisanu F., Garribba E., Merlino A. (2023). Interaction of V^IV^O-8-hydroxyquinoline species with RNase A: The effect of metal ligands in the protein adduct stabilization. Inorg. Chem. Front..

[22] Tito G., Ferraro G., Pisanu F., Garribba E., Merlino A. (2024). Non-Covalent and Covalent Binding of New Mixed-Valence Cage-like Polyoxidovanadate Clusters to Lysozyme. Angew. Chem., Int. Ed..

[23] Dinda R., Garribba E., Sanna D., Crans D.C., Pessoa J.C. (2025). Hydrolysis, Ligand Exchange, and Redox Properties of Vanadium Compounds: Implications of Solution Transformation on Biological, Therapeutic, and Environmental Applications. Chem. Rev..

[24] Zhang Y., Watanabe S., Tsutsumi A., Kadokura H., Kikkawa M., Inaba K. (2021). Cryo-EM Analysis Provides New Mechanistic Insight into ATP Binding to Ca^2+^-ATPase SERCA2b. EMBO J..

[25] Aureliano M., Fraqueza G., Berrocal M., Córdoba Granados J.J., Gumerova N.I., Rompel A., Gutiérrez Merino C., Mata A.M. (2022). Inhibition of SERCA and PMCA Ca^2+^-ATPase Activities by Polyoxotungstates. J. Inorg. Biochem..

[26] Fraqueza G., Ohlin C.A., Casey W.H., Aureliano M. (2012). Sarcoplasmic Reticulum Calcium ATPase Interactions with Decaniobate, Decavanadate, Vanadate, Tungstate and Molybdate. J. Inorg. Biochem..

[27] Poejo J., Gumerova N.I., Rompel A., Mata A.M., Aureliano M., Gutierrez-Merino C. (2024). Unveiling the Agonistic Properties of Preyssler-Type Polyoxotungstate s on Purinergic P2 Receptors. J. Inorg. Biochem..

[28] Himeno S., Kawasaki K., Hashimoto M. (2008). Preparation and Characterization of an α-Wells–Dawson-Type [V^2^Mo_18_O_62_]^6−^ Complex. Bull. Chem. Soc. Jpn..

[29] Baur W.H. (1974). The geometry of polyhedral distortions. Predictive relationships for the phosphate group. Acta Crystallogr. B.

[30] Sun Z., Yu X., Zhen Q., Yu T., Wang X., Yang G., Song Y., Pang H. (2025). Synthesis of Two New Polyoxometalate-Based Organic Complexes from 2D to 3D Structures for Improving Supercapacitor Performance. Dalton Trans..

[31] Ma X., Jing Z., Li K., Chen Y., Li D., Ma P., Wang J., Niu J. (2022). Copper-Containing Polyoxometalate-Based Metal−Organic Framework as a Catalyst for the Oxidation of Silanes: Effective Cooperative Catalysis by Metal Sites and POM Precursor. Inorg. Chem..

[32] Brown I.D., Altermatt D. (1985). Bond-valence parameters obtained from a systematic analysis of the Inorganic Crystal Structure Database. Acta Crystallogr. B.

[33] Ishida H., Kashino S. (2000). Ethylammonium and Diethylammonium Salts of Chloranilic Acid. Cryst. Struct. Commun..

[34] Akouibaa M., Slassi S., Direm A., Nasif V., Sayin K., Cruciani G., Precisvalle N., Lachkar M., El Bali B. (2025). mer-[Ni(dien)_2_]Cl_2_·H_2_O and mer-[Ni(dien)_2_](NO_3_)_2_ Complexes (dien = Diethylenetriamine): Synthesis, Physicochemical and Computational Studies, Antioxidant Activity, and Their Use as Pre-cursors for Ni/NiO Nanoparticle Preparation. J. Mol. Struct..

[35] Maalaoui A., Agwamba E.C., Louis H., Mathias G.E., Rzaigui M., Akriche S. (2023). Combined Experimental and Computational Study of V-Substituted Lindqvist Polyoxotungstate: Screening by Docking for Potential Antidiabetic Activity. Inorg. Chem..

[36] Humelnicu D., Olariu R.-I., Sandu I., Apostolescu N., Sandu A.V., Arsene C. (2008). New Heteropolyoxotungstates and Heteropolyoxomolybdates Containing Radioactive Ions (uranyl and thorium) in their Structure. Rev. Chim..

[37] Téllez C.A., Hollauer E., Mondragon M.A., Castaño V.M. (2001). Fourier transform infrared and Raman spectra, vibrational assignment and ab initio calculations of terephthalic acid and related compounds. Spectrochim. Acta A Mol. Biomol. Spectrosc..

[38] Uchida S., Kamata K., Ogasawara Y., Fujita M., Mizuno N. (2012). Structural and dynamical aspects of alkylammonium salts of a silicodecatungstate as heterogeneous epoxidation catalysts. Dalton Trans..

[39] Wang C., Zhou J., Chu L., Zhang M., Xu C., Liu J., Li S. (2024). Diethylenetriamine-Functionalized Reduced Graphene Oxide Having More Amino Groups for Methylene Blue Removal. RSC Adv..

[40] Fonseca C., Fraqueza G., Carabineiro S.A.C., Aureliano M. (2020). The Ca^2+^-ATPase Inhibition Potential of Gold(I, III) Compounds. Inorganics.

[41] Yatime L., Buch-Pedersen M.J., Musgaard M., Morth J.P., Winther A.-M.L., Pedersen B.P., Olesen C., Andersen J.P., Vilsen B., Schiøtt B. (2009). P-Type ATPases as Drug Targets: Tools for Medicine and Science. Biochim. Biophys. Acta-Bioenerg..

[42] Aureliano M., Gumerova N.I., Sciortino G., Garribba E., Rompel A., Crans D.C. (2021). Polyoxovanadates with emerging biomedical activities. Coord. Chem. Rev..

[43] Brini M., Calì T., Ottolini D., Carafoli E. (2014). Neuronal calcium signaling: Function and dysfunction. Cell. Mol. Life Sci..

[44] Joshi M., Joshi S., Khambete M., Degani M. (2023). Role of calcium dysregulation in Alzheimer’s disease and its therapeutic implications. Chem. Biol. Drug Des..

[45] Ge M., Zhang J., Chen S., Huang Y., Chen W., He L., Zhang Y. (2020). Role of Calcium Homeostasis in Alzheimer’s Disease. Neuropsychiatr. Dis. Treat..

[46] Boczek T., Sobolczyk M., Mackiewicz J., Lisek M., Ferenc B., Guo F., Zylinska L. (2021). Crosstalk among Calcium ATPases: PMCA, SERCA and SPCA in Mental Diseases. Int. J. Mol. Sci..

[47] Mata A.M., Berrocal M., Marcos D., Sepúlveda M.R. (2010). Impairment of PMCA Activity by Amyloid β-Peptide in Membranes from Alzheimer's Disease-Affected Brain and from Other Model Systems. Biophys. J..

[48] Drews A., Flint J., Shivji N., Jönsson P., Wirthensohn D., De Genst E., Vincke C., Muyldermans S., Dobson C., Klenerman D. (2016). Individual aggregates of amyloid beta induce temporary calcium influx through the cell membrane of neuronal cells. Sci. Rep..

[49] Fraqueza G., Fuentes J., Krivosudský L., Dutta S., Mal S.S., Roller A., Giester G., Rompel A., Aureliano M. (2019). Inhibition of Na^+^/K^+^- and Ca^2+^-ATPase Activities by Phosphotetradecavanadate. J. Inorg. Biochem..

[50] Solé-Daura A., Poblet J.M., Carbó J.J. (2020). Structure–Activity Relationships for the Affinity of Chaotropic Polyoxometalate Anions towards Proteins. Chem. Eur. J..

[51] Sciortino G., Aureliano M., Garribba E. (2021). Rationalizing the Decavanadate(V) and Oxidovanadium(IV) Binding to G-Actin and the Competition with Decaniobate(V) and ATP. Inorg. Chem..

[52] Gumerova N.I., Al-Sayed E., Krivosudský L., Čipčić-Paljetak H., Verbanac D., Rompel A. (2018). Antibacterial Activity of Polyoxometalates Against Moraxella catarrhalis. Sect. Inorg. Chem..

[53] Čolović M.B., Bajuk-Bogdanović D.V., Avramović N.S., Holclajtner-Antunović I.D., Bošnjaković-Pavlović N.S., Vasić V.M., Krstić D.Z. (2011). Inhibition of rat synaptic membrane Na^+^/K^+^-ATPase and ecto-nucleoside triphosphate diphosphohydrolases by 12-tungstosilicic and 12-tungstophosphoric acid. Bioorganic Med. Chem..

[54] Krstić D., Čolović M., Bosnjaković-Pavlović N., Spasojević-De Bire A., Vasić V. (2009). Influence of decavanadate on rat synaptic plasma membrane ATPases activity. Gen. Physiol. Biophys..

[55] Brito B.R., Camilo H.d.S., Cruz A.F.d., Ribeiro R.R., de Sá E.L., Camargo de Oliveira C., Fraqueza G., Klassen G., Aureliano M., Nunes G.G. (2025). Mixed-Valence Pentadecavanadate with Ca^2+^-ATPase Inhibition Potential and Anti-Breast Cancer Activity. Inorganics.

[56] Bijelic A., Aureliano M., Rompel A. (2018). The antibacterial activity of polyoxometalates: Structures, antibiotic effects and future perspectives. Chem. Commun..

[57] Carvalho F., Aureliano M. (2023). Polyoxometalates Impact as Anticancer Agents. Int. J. Mol. Sci..

[58] Gao N., Liu Z., Zhang H., Liu C., Yu D., Ren J., Qu X. (2022). Site-Directed Chemical Modification of Amyloid by Polyoxometalates for Inhibition of Protein Misfolding and Aggregation. Angew. Chem. Int. Ed. Engl..

[59] Atrian-Blasco E., de Cremoux L., Lin X., Mitchell-Heggs R., Sabater L., Blanchard S., Hureau C. (2022). Keggin-type polyoxometalates as Cu(II) chelators in the context of Alzheimer’s disease. Chem. Commun..

[60] Zaken B., Bouhnik K., Omer R.N., Bloch N., Samson A.O. (2025). Polyoxometalates bind multiple targets involved in Alzheimer’s disease. J. Biol. Inorg. Chem..

[61] Dan K., Yeh H.-L. (2024). Biological Activity of Polyoxometalates and Their Applications in Anti-Aging. Med. Res. Arch..

[62] Sheldrick G.M. (2008). A short history of SHELX. Acta Crystallogr. A.

[63] Sheldrick G.M. (2015). Crystal structure refinement with SHELXL. Acta Crystallogr. C.

