# Supplementary material for: Dawson- and Lindqvist-Type Hybrid Polyoxometalates: Synthesis, Characterization and Ca2+-ATPase Inhibition Potential
Source: Molecules. 2025 Nov 7;30(22):4334. doi: 10.3390/molecules30224334 (PMC12654597; doi:10.3390/molecules30224334)
Supplement: Supplementary file 1 [file molecules-30-04334-s001.zip › molecules-3838746-supplementary.pdf]

Supplementary information

Dawson- and Lindqvist-Type Hybrid Polyoxometalates: Synthesis, Characterization and Ca^{2+}-ATPase Inhibition Potential

Islem Meskini[1]*, Frédéric Capet[2], Gil Fraqueza[3,4], Necmi Dege[5], Muhammad Nawaz Tahir[6], Brahim Ayed[1], Manuel Aureliano[4,7]*

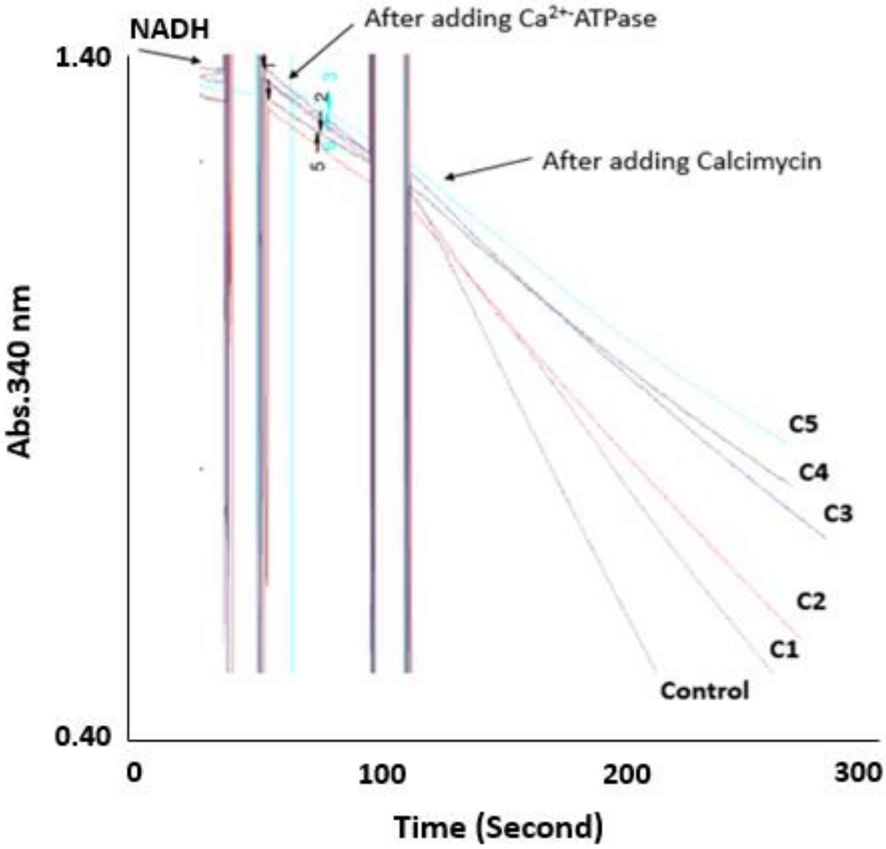

Figure S1: Example of experimental registration of the Ca^{2+}-ATPase inhibition by POMs, using the coupled enzymatic assay. In the absence of the POM the control assay was obtained that is a kinetic with a higher slope, while the by increasing the concentrations of the POMs lower enzymatic kinetic slopes were found due to the inhibition of the ATPase activity. C1, C2, C3, C4 and C5, means increasing concentrations of compound 1 or compound 2.

Table S1: Selected bond lengths (Å) and angles (°) for $(C_2H_8N)_6[V_2Mo_{18}O_{62}].3H_2O$

Parameters	X-Ray	Parameters	X-Ray
	**Bond length (Å)**		
V1—O1	1.686 (4)	Mo10—O28	1.899 (5)
V1—O48	1.692 (4)	Mo10—O58	1.994 (5)
V1—O55	1.695 (4)	Mo10—O49	2.023 (5)
V1—O23	1.755 (4)	Mo10—O23	2.300 (4)
V2—O18	1.690 (4)	Mo11—O9	1.687 (5)
V2—O5	1.691 (4)	Mo11—O58	1.864 (4)
V2—O2	1.694 (4)	Mo11—O22	1.883 (5)
V2—O39	1.759 (4)	Mo11—O57	1.950 (5)

Mo1—O46	1.695 (5)	Mo11—O36	2.014 (5)
Mo1—O14	1.788 (4)	Mo11—O48	2.308 (4)
Mo1—O17	1.877 (5)	Mo12—O16	1.675 (5)
Mo1—O32	1.967 (5)	Mo12—O6	1.833 (5)
Mo1—O11	2.097 (5)	Mo12—O21	1.872 (5)
Mo1—O5	2.269 (4)	Mo12—O25	1.969 (4)
Mo2—O62	1.698 (5)	Mo12—O34	2.066 (5)
Mo2—O31	1.821 (5)	Mo12—O18	2.286 (4)
Mo2—O25	1.849 (4)	Mo13—O38	1.694 (5)
Mo2—O37	1.984 (4)	Mo13—O36	1.831 (5)
Mo2—O20	2.096 (5)	Mo13—O41	1.864 (5)
Mo2—O2	2.247 (4)	Mo13—O21	1.962 (5)
Mo3—O40	1.697 (5)	Mo13—O35	2.073 (5)
Mo3—O27	1.837 (5)	Mo13—O18	2.281 (4)
Mo3—O7	1.888 (4)	Mo14—O44	1.678 (5)
Mo3—O53	1.950 (4)	Mo14—O26	1.799 (5)
Mo3—O31	2.053 (5)	Mo14—O13	1.856 (5)
Mo3—O55	2.262 (4)	Mo14—O59	2.010 (4)
Mo4—O33	1.680 (5)	Mo14—O28	2.060 (5)
Mo4—O10	1.804 (5)	Mo14—O23	2.338 (4)
Mo4—O49	1.902 (5)	Mo15—O19	1.676 (5)
Mo4—O13	1.993 (5)	Mo15—O3	1.822 (4)
Mo4—O27	2.002 (5)	Mo15—O32	1.901 (5)
Mo4—O23	2.291 (4)	Mo15—O41	1.937 (5)
Mo5—O15	1.694 (5)	Mo15—O50	2.067 (5)
Mo5—O50	1.794 (5)	Mo15—O5	2.286 (4)
Mo5—O57	1.865 (5)	Mo16—O56	1.695 (4)
Mo5—O8	1.981 (5)	Mo16—O47	1.811 (5)
Mo5—O12	2.084 (4)	Mo16—O42	1.845 (4)
Mo5—O1	2.282 (4)	Mo16—O7	2.006 (4)
Mo6—O30	1.683 (5)	Mo16—O26	2.103 (5)
Mo6—O59	1.819 (4)	Mo16—O55	2.273 (4)
Mo6—O8	1.882 (5)	Mo17—O61	1.695 (5)
Mo6—O42	1.956 (4)	Mo17—O35	1.817 (5)
Mo6—O14	2.063 (4)	Mo17—O45	1.856 (5)
Mo6—O1	2.304 (4)	Mo17—O3	2.010 (5)

Mo7—O43	1.681 (5)	Mo17—O29	2.019 (5)
Mo7—O11	1.795 (5)	Mo17—O39	2.300 (4)
Mo7—O29	1.865 (5)	Mo18—O52	1.685 (5)
Mo7—O4	2.027 (5)	Mo18—O4	1.833 (5)
Mo7—O51	2.057 (5)	Mo18—O37	1.885 (4)
Mo7—O39	2.323 (4)	Mo18—O17	1.933 (4)
Mo8—O24	1.701 (5)	Mo18—O47	2.071 (5)
Mo8—O34	1.804 (4)	Mo18—O2	2.302 (4)
Mo8—O53	1.850 (5)	N1—C1	1.4852 (1)
Mo8—O22	1.984 (5)	N1—C2	1.4934 (1)
Mo8—O10	2.107 (5)	N2—C4	1.4824 (1)
Mo8—O48	2.237 (4)	N2—C3	1.5005 (1)
Mo9—O60	1.689 (5)	N3—C6	1.1012 (1)
Mo9—O20	1.808 (5)	N3—C5	1.4208 (1)
Mo9—O51	1.871 (5)	N4—C7	1.4506 (1)
Mo9—O6	1.995 (5)	N4—C8	1.5858 (1)
Mo9—O45	2.018 (5)	N5—C10	1.4019 (1)
Mo9—O39	2.305 (4)	N5—C9	1.5810 (1)
Mo10—O54	1.677 (5)	N6—C11	1.4799 (1)
Mo10—O12	1.803 (5)	N6—C12	1.5035 (1)

Bond angles (°)

O1—V1—O48	111.6 (2)	O16—Mo12—O6	101.5 (2)
O1—V1—O55	112.3 (2)	O16—Mo12—O21	101.0 (2)
O48—V1—O55	112.4 (2)	O6—Mo12—O21	98.2 (2)
O1—V1—O23	106.9 (2)	O16—Mo12—O25	102.2 (2)
O48—V1—O23	106.4 (2)	O6—Mo12—O25	86.88 (19)
O55—V1—O23	106.6 (2)	O21—Mo12—O25	154.78 (19)
O18—V2—O5	111.3 (2)	O16—Mo12—O34	92.3 (2)
O18—V2—O2	111.9 (2)	O6—Mo12—O34	163.84 (18)
O5—V2—O2	112.3 (2)	O21—Mo12—O34	87.33 (18)
O18—V2—O39	107.2 (2)	O25—Mo12—O34	81.93 (18)
O5—V2—O39	106.9 (2)	O16—Mo12—O18	170.3 (2)
O2—V2—O39	106.9 (2)	O6—Mo12—O18	87.11 (17)
O46—Mo1—O14	101.4 (2)	O21—Mo12—O18	73.00 (17)
O46—Mo1—O17	102.8 (2)	O25—Mo12—O18	82.66 (17)
O14—Mo1—O17	96.0 (2)	O34—Mo12—O18	79.95 (16)

O46—Mo1—O32	97.8 (2)	O38—Mo13—O36	100.2 (2)
O14—Mo1—O32	94.5 (2)	O38—Mo13—O41	104.4 (2)
O17—Mo1—O32	154.46 (19)	O36—Mo13—O41	95.0 (2)
O46—Mo1—O11	91.7 (2)	O38—Mo13—O21	97.8 (2)
O14—Mo1—O11	166.78 (19)	O36—Mo13—O21	92.5 (2)
O17—Mo1—O11	81.98 (19)	O41—Mo13—O21	154.88 (19)
O32—Mo1—O11	82.49 (19)	O38—Mo13—O35	93.3 (2)
O46—Mo1—O5	166.9 (2)	O36—Mo13—O35	166.31 (19)
O14—Mo1—O5	87.35 (18)	O41—Mo13—O35	83.32 (19)
O17—Mo1—O5	85.69 (17)	O21—Mo13—O35	83.77 (19)
O32—Mo1—O5	71.61 (17)	O38—Mo13—O18	168.2 (2)
O11—Mo1—O5	79.48 (17)	O36—Mo13—O18	85.60 (17)
O62—Mo2—O31	100.9 (2)	O41—Mo13—O18	85.10 (17)
O62—Mo2—O25	104.1 (2)	O21—Mo13—O18	71.59 (16)
O31—Mo2—O25	96.2 (2)	O35—Mo13—O18	80.73 (16)
O62—Mo2—O37	96.5 (2)	O44—Mo14—O26	105.7 (2)
O31—Mo2—O37	93.15 (19)	O44—Mo14—O13	100.6 (2)
O25—Mo2—O37	155.27 (19)	O26—Mo14—O13	98.5 (2)
O62—Mo2—O20	90.9 (2)	O44—Mo14—O59	102.2 (2)
O31—Mo2—O20	167.89 (19)	O26—Mo14—O59	86.2 (2)
O25—Mo2—O20	83.71 (19)	O13—Mo14—O59	154.59 (19)
O37—Mo2—O20	82.40 (18)	O44—Mo14—O28	95.9 (2)
O62—Mo2—O2	166.2 (2)	O26—Mo14—O28	156.32 (19)
O31—Mo2—O2	87.39 (18)	O13—Mo14—O28	86.9 (2)
O25—Mo2—O2	85.77 (18)	O59—Mo14—O28	79.70 (19)
O37—Mo2—O2	71.83 (17)	O44—Mo14—O23	166.9 (2)
O20—Mo2—O2	80.52 (16)	O26—Mo14—O23	87.14 (17)
O40—Mo3—O27	101.0 (2)	O13—Mo14—O23	74.19 (17)
O40—Mo3—O7	101.8 (2)	O59—Mo14—O23	81.14 (17)
O27—Mo3—O7	97.5 (2)	O28—Mo14—O23	72.06 (17)
O40—Mo3—O53	100.6 (2)	O19—Mo15—O3	102.0 (2)
O27—Mo3—O53	87.74 (19)	O19—Mo15—O32	100.2 (2)
O7—Mo3—O53	155.51 (19)	O3—Mo15—O32	97.3 (2)
O40—Mo3—O31	91.5 (2)	O19—Mo15—O41	102.7 (2)
O27—Mo3—O31	165.48 (19)	O3—Mo15—O41	88.6 (2)
O7—Mo3—O31	86.95 (19)	O32—Mo15—O41	154.61 (19)

O53—Mo3—O31	82.75 (18)	O19—Mo15—O50	91.5 (2)
O40—Mo3—O55	171.0 (2)	O3—Mo15—O50	164.97 (19)
O27—Mo3—O55	87.39 (18)	O32—Mo15—O50	86.43 (19)
O7—Mo3—O55	73.59 (17)	O41—Mo15—O50	82.12 (18)
O53—Mo3—O55	82.81 (17)	O19—Mo15—O5	168.4 (2)
O31—Mo3—O55	80.58 (16)	O3—Mo15—O5	87.93 (18)
O33—Mo4—O10	104.7 (2)	O32—Mo15—O5	72.33 (17)
O33—Mo4—O49	100.3 (3)	O41—Mo15—O5	83.28 (17)
O10—Mo4—O49	95.7 (2)	O50—Mo15—O5	79.30 (16)
O33—Mo4—O13	96.4 (2)	O56—Mo16—O47	100.8 (2)
O10—Mo4—O13	157.9 (2)	O56—Mo16—O42	104.5 (2)
O49—Mo4—O13	86.7 (2)	O47—Mo16—O42	96.9 (2)
O33—Mo4—O27	100.4 (2)	O56—Mo16—O7	97.1 (2)
O10—Mo4—O27	85.9 (2)	O47—Mo16—O7	92.13 (19)
O49—Mo4—O27	158.0 (2)	O42—Mo16—O7	154.53 (19)
O13—Mo4—O27	83.92 (19)	O56—Mo16—O26	91.9 (2)
O33—Mo4—O23	168.6 (2)	O47—Mo16—O26	166.92 (18)
O10—Mo4—O23	86.34 (18)	O42—Mo16—O26	82.78 (18)
O49—Mo4—O23	75.38 (19)	O7—Mo16—O26	83.15 (18)
O13—Mo4—O23	72.99 (17)	O56—Mo16—O55	166.4 (2)
O27—Mo4—O23	82.88 (17)	O47—Mo16—O55	87.04 (17)
O15—Mo5—O50	101.4 (2)	O42—Mo16—O55	85.38 (17)
O15—Mo5—O57	103.4 (2)	O7—Mo16—O55	71.31 (16)
O50—Mo5—O57	96.4 (2)	O26—Mo16—O55	79.90 (16)
O15—Mo5—O8	97.4 (2)	O61—Mo17—O35	104.8 (2)
O50—Mo5—O8	93.9 (2)	O61—Mo17—O45	100.3 (2)
O57—Mo5—O8	154.43 (19)	O35—Mo17—O45	96.6 (2)
O15—Mo5—O12	91.8 (2)	O61—Mo17—O3	101.0 (2)
O50—Mo5—O12	166.67 (19)	O35—Mo17—O3	85.2 (2)
O57—Mo5—O12	82.31 (19)	O45—Mo17—O3	157.4 (2)
O8—Mo5—O12	82.38 (19)	O61—Mo17—O29	95.6 (2)
O15—Mo5—O1	167.0 (2)	O35—Mo17—O29	157.7 (2)
O50—Mo5—O1	86.91 (18)	O45—Mo17—O29	88.5 (2)
O57—Mo5—O1	85.34 (18)	O3—Mo17—O29	82.0 (2)
O8—Mo5—O1	71.86 (17)	O61—Mo17—O39	167.9 (2)
O12—Mo5—O1	79.76 (16)	O35—Mo17—O39	87.14 (18)

O30—Mo6—O59	102.4 (2)	O45—Mo17—O39	75.59 (18)
O30—Mo6—O8	100.6 (2)	O3—Mo17—O39	82.06 (17)
O59—Mo6—O8	97.3 (2)	O29—Mo17—O39	73.06 (18)
O30—Mo6—O42	102.8 (2)	O52—Mo18—O4	101.3 (2)
O59—Mo6—O42	88.30 (19)	O52—Mo18—O37	100.8 (2)
O8—Mo6—O42	154.15 (19)	O4—Mo18—O37	96.2 (2)
O30—Mo6—O14	91.8 (2)	O52—Mo18—O17	102.9 (2)
O59—Mo6—O14	163.98 (19)	O4—Mo18—O17	88.9 (2)
O8—Mo6—O14	87.13 (19)	O37—Mo18—O17	154.26 (19)
O42—Mo6—O14	81.34 (18)	O52—Mo18—O47	92.4 (2)
O30—Mo6—O1	168.84 (19)	O4—Mo18—O47	165.11 (19)
O59—Mo6—O1	87.62 (18)	O37—Mo18—O47	86.91 (19)
O8—Mo6—O1	73.01 (17)	O17—Mo18—O47	82.23 (18)
O42—Mo6—O1	82.08 (17)	O52—Mo18—O2	169.4 (2)
O14—Mo6—O1	78.92 (16)	O4—Mo18—O2	87.51 (18)
O43—Mo7—O11	103.3 (2)	O37—Mo18—O2	72.23 (17)
O43—Mo7—O29	102.0 (3)	O17—Mo18—O2	82.86 (17)
O11—Mo7—O29	98.8 (2)	O47—Mo18—O2	79.53 (16)
O43—Mo7—O4	99.8 (2)	V1—O1—Mo5	126.8 (2)
O11—Mo7—O4	85.5 (2)	V1—O1—Mo6	122.4 (2)
O29—Mo7—O4	156.1 (2)	Mo5—O1—Mo6	94.26 (16)
O43—Mo7—O51	98.5 (2)	V2—O2—Mo2	126.4 (2)
O11—Mo7—O51	155.64 (19)	V2—O2—Mo18	122.4 (2)
O29—Mo7—O51	87.1 (2)	Mo2—O2—Mo18	95.27 (16)
O4—Mo7—O51	80.05 (19)	Mo15—O3—Mo17	150.0 (3)
O43—Mo7—O39	170.0 (2)	Mo18—O4—Mo7	148.8 (3)
O11—Mo7—O39	86.67 (18)	V2—O5—Mo1	126.4 (2)
O29—Mo7—O39	75.20 (18)	V2—O5—Mo15	122.6 (2)
O4—Mo7—O39	81.65 (17)	Mo1—O5—Mo15	95.24 (16)
O51—Mo7—O39	71.95 (17)	Mo12—O6—Mo9	152.4 (3)
O24—Mo8—O34	101.6 (2)	Mo3—O7—Mo16	119.4 (2)
O24—Mo8—O53	104.5 (2)	Mo6—O8—Mo5	120.9 (2)
O34—Mo8—O53	96.5 (2)	Mo4—O10—Mo8	152.4 (3)
O24—Mo8—O22	95.1 (2)	Mo7—O11—Mo1	155.1 (3)
O34—Mo8—O22	93.2 (2)	Mo10—O12—Mo5	153.0 (3)
O53—Mo8—O22	155.8 (2)	Mo14—O13—Mo4	120.3 (2)

O24—Mo8—O10	90.0 (2)	Mo1—O14—Mo6	161.4 (3)
O34—Mo8—O10	168.1 (2)	Mo1—O17—Mo18	154.5 (3)
O53—Mo8—O10	83.27 (19)	V2—O18—Mo13	126.2 (2)
O22—Mo8—O10	82.72 (19)	V2—O18—Mo12	122.5 (2)
O24—Mo8—O48	164.9 (2)	Mo13—O18—Mo12	94.07 (15)
O34—Mo8—O48	87.72 (18)	Mo9—O20—Mo2	152.6 (3)
O53—Mo8—O48	86.00 (18)	Mo12—O21—Mo13	121.3 (2)
O22—Mo8—O48	72.29 (18)	Mo11—O22—Mo8	120.3 (2)
O10—Mo8—O48	80.36 (17)	V1—O23—Mo4	123.2 (2)
O60—Mo9—O20	103.8 (2)	V1—O23—Mo10	122.6 (2)
O60—Mo9—O51	102.1 (2)	Mo4—O23—Mo10	93.98 (16)
O20—Mo9—O51	96.0 (2)	V1—O23—Mo14	122.5 (2)
O60—Mo9—O6	100.3 (2)	Mo4—O23—Mo14	92.33 (15)
O20—Mo9—O6	85.26 (19)	Mo10—O23—Mo14	94.13 (16)
O51—Mo9—O6	156.56 (19)	Mo2—O25—Mo12	153.7 (3)
O60—Mo9—O45	97.0 (2)	Mo14—O26—Mo16	153.2 (3)
O20—Mo9—O45	157.11 (19)	Mo3—O27—Mo4	150.2 (3)
O51—Mo9—O45	88.8 (2)	Mo10—O28—Mo14	118.1 (2)
O6—Mo9—O45	81.71 (19)	Mo7—O29—Mo17	118.9 (2)
O60—Mo9—O39	169.3 (2)	Mo2—O31—Mo3	158.8 (3)
O20—Mo9—O39	86.86 (18)	Mo15—O32—Mo1	120.8 (2)
O51—Mo9—O39	75.56 (18)	Mo8—O34—Mo12	159.4 (3)
O6—Mo9—O39	81.16 (17)	Mo17—O35—Mo13	153.6 (3)
O45—Mo9—O39	72.63 (17)	Mo13—O36—Mo11	161.4 (3)
O54—Mo10—O12	102.9 (3)	Mo18—O37—Mo2	120.7 (2)
O54—Mo10—O28	100.7 (2)	V2—O39—Mo17	123.4 (2)
O12—Mo10—O28	95.5 (2)	V2—O39—Mo9	123.1 (2)
O54—Mo10—O58	99.5 (2)	Mo17—O39—Mo9	92.87 (15)
O12—Mo10—O58	86.9 (2)	V2—O39—Mo7	122.5 (2)
O28—Mo10—O58	158.6 (2)	Mo17—O39—Mo7	92.76 (15)
O54—Mo10—O49	97.1 (3)	Mo9—O39—Mo7	93.82 (15)
O12—Mo10—O49	158.7 (2)	Mo13—O41—Mo15	153.4 (3)
O28—Mo10—O49	87.8 (2)	Mo16—O42—Mo6	154.8 (3)
O58—Mo10—O49	82.7 (2)	Mo17—O45—Mo9	118.9 (2)
O54—Mo10—O23	169.5 (2)	Mo16—O47—Mo18	159.0 (3)
O12—Mo10—O23	87.32 (18)	V1—O48—Mo8	126.1 (2)

O28—Mo10—O23	75.72 (18)	V1—O48—Mo11	123.8 (2)
O58—Mo10—O23	83.14 (17)	Mo8—O48—Mo11	95.07 (16)
O49—Mo10—O23	73.06 (18)	Mo4—O49—Mo10	117.6 (2)
O9—Mo11—O58	100.7 (2)	Mo5—O50—Mo15	160.2 (3)
O9—Mo11—O22	101.8 (2)	Mo9—O51—Mo7	118.7 (2)
O58—Mo11—O22	95.2 (2)	Mo8—O53—Mo3	154.4 (3)
O9—Mo11—O57	102.5 (2)	V1—O55—Mo3	123.5 (2)
O58—Mo11—O57	86.7 (2)	V1—O55—Mo16	126.2 (2)
O22—Mo11—O57	154.80 (19)	Mo3—O55—Mo16	95.70 (16)
O9—Mo11—O36	93.4 (2)	Mo5—O57—Mo11	156.1 (3)
O58—Mo11—O36	164.1 (2)	Mo11—O58—Mo10	149.3 (3)
O22—Mo11—O36	88.96 (19)	Mo6—O59—Mo14	150.1 (3)
O57—Mo11—O36	83.06 (19)	C1—N1—C2	114.038 (2)
O9—Mo11—O48	171.3 (2)	C4—N2—C3	111.587 (3)
O58—Mo11—O48	86.47 (18)	C6—N3—C5	135.031 (2)
O22—Mo11—O48	72.38 (18)	C7—N4—C8	106.421 (2)
O57—Mo11—O48	82.70 (17)	C10—N5—C9	113.023 (3)
O36—Mo11—O48	80.15 (16)	C11—N6—C12	109.997 (1)

Table S2: Selected bond lengths (Å) and angles (°) for $(C_4H_{16}N_3)_2(C_4H_{16}N_3)_4[V_2W_4O_{19}]_3.12H_2O$.

**Parameters**	**X-Ray**	**Parameters**	**X-Ray**
	**Bond length (Å)**		
W1—O1	1.711 (11)	W9—O22	1.918 (11)
W1—O2	1.912 (11)	W9—O27	1.975 (11)
W1—O5	1.926 (11)	W9—O25	2.2967 (9)
W1—O4	1.936 (10)	V7—O20	1.638 (12)
W1—O3	1.950 (11)	V7—O23	1.890 (11)
W1—O6	2.295 (10)	V7—O22	1.902 (11)
W1—W2	3.2283 (14)	V7—O24	1.953 (11)
W2—O7	1.664 (11)	V7—O21	1.959 (11)
W2—O2	1.910 (11)	V7—O25	2.3096 (10)
W2—O9	1.929 (11)	V8—O26	1.678 (12)
W2—O8	1.941 (11)	V8—O21	1.902 (11)

W2—O10	1.946 (11)	V8—O27	1.906 (11)
W2—O6	2.310 (11)	V8—O23^{i}	1.917 (11)
W2—W3	3.2335 (15)	V8—O28^{i}	1.919 (10)
W3—O11	1.660 (12)	V8—O25	2.2976 (9)
W3—O8	1.881 (11)	V9—O29	1.658 (11)
W3—O13	1.892 (12)	V9—O28	1.898 (11)
W3—O3	1.918 (10)	V9—O24^{i}	1.906 (10)
W3—O12	1.969 (11)	V9—O22	1.918 (11)
W3—O6	2.305 (11)	V9—O27	1.975 (11)
W4—O14	1.663 (12)	V9—O25	2.2967 (9)
W4—O15	1.891 (12)	O30—H30A	0.840 (10)
W4—O16	1.915 (11)	O30—H30B	0.840 (10)
W4—O4	1.935 (10)	O31—H31A	0.7122 (1)
W4—O9	1.959 (11)	O31—H31B	0.7614 (1)
W4—O6	2.326 (11)	O32—H32A	0.851 (10)
W4—W5	3.1945 (15)	O32—H32B	0.850 (10)
W5—O17	1.641 (11)	O33—H33A	0.871 (10)
W5—O15	1.888 (12)	O33—H33B	0.870 (10)
W5—O18	1.897 (11)	O34—H34A	0.95 (2)
W5—O5	1.917 (11)	O34—H34B	0.95 (2)
W5—O12	1.972 (11)	O35A—H35A	0.870 (10)
W5—O6	2.282 (11)	O35A—H35B	0.870 (11)
W5—W6	3.2374 (15)	O35B—H35C	0.870 (11)
W6—O19	1.660 (12)	O35B—H35D	0.870 (10)
W6—O18	1.911 (11)	N1—C1	1.48 (2)
W6—O16	1.915 (11)	N1—H1A	0.849 (10)
W6—O10	1.946 (11)	N1—H1B	0.850 (10)
W6—O13	1.948 (11)	N1—H1C	0.849 (10)
W6—O6	2.311 (10)	N2—C2	1.49 (2)
V1—O1	1.711 (11)	N2—C3	1.53 (2)
V1—O2	1.912 (11)	N2—H2A	0.820 (10)
V1—O5	1.926 (11)	N2—H2B	0.820 (10)
V1—O4	1.936 (10)	N3—C4	1.48 (2)
V1—O3	1.950 (11)	N3—H3A	0.8900
V1—O6	2.295 (10)	N3—H3B	0.8900
V2—O7	1.664 (11)	N3—H3C	0.8900

V2—O2	1.910 (11)	C1—C2	1.49 (2)
V2—O9	1.929 (11)	C1—H1D	0.9700
V2—O8	1.941 (11)	C1—H1E	0.9700
V2—O10	1.946 (11)	C2—H2C	0.9700
V2—O6	2.310 (11)	C2—H2D	0.9700
V3—O11	1.660 (12)	C3—C4	1.45 (2)
V3—O8	1.881 (11)	C3—H3D	0.9700
V3—O13	1.892 (12)	C3—H3E	0.9700
V3—O3	1.918 (10)	C4—H4A	0.9700
V3—O12	1.969 (11)	C4—H4B	0.9700
V3—O6	2.305 (11)	N4—C5	1.48 (2)
V5—O17	1.641 (11)	N4—H4C	0.8900
V5—O15	1.888 (12)	N4—H4D	0.8900
V5—O18	1.897 (11)	N4—H4E	0.8900
V5—O5	1.917 (11)	N5—C6	1.46 (2)
V5—O12	1.972 (11)	N5—C7	1.52 (2)
V5—O6	2.282 (11)	N5—H5A	0.821 (11)
W7—O20	1.638 (12)	N5—H5B	0.820 (10)
W7—O23	1.890 (11)	N6—C8	1.46 (2)
W7—O22	1.902 (11)	N6—H6A	0.8900
W7—O24	1.953 (11)	N6—H6B	0.8900
W7—O21	1.959 (11)	N6—H6C	0.8900
W7—O25	2.3096 (10)	C5—C6	1.52 (2)
W8—O26	1.678 (12)	C5—H5C	0.9700
W8—O21	1.902 (11)	C5—H5D	0.9700
W8—O27	1.906 (11)	C6—H6D	0.9700
W8—O23^{i}	1.917 (11)	C6—H6E	0.9700
W8—O28^{i}	1.919 (10)	C7—C8	1.51 (2)
W8—O25	2.2976 (9)	C7—H7A	0.9700
W8—W9^{i}	3.2326 (13)	C7—H7B	0.9700
W9—O29	1.658 (11)	C8—H8A	0.9700
W9—O28	1.898 (11)	C8—H8B	0.9700
W9—O24^{i}	1.906 (10)		
	**Bond angles (°)**		
O1—W1—O2	101.8 (5)	V3—O8—V2	115.5 (6)
O1—W1—O5	103.9 (5)	W2—O9—W4	116.0 (5)

O2—W1—O5	154.3 (5)	W2—O10—W6	115.0 (5)
O1—W1—O4	103.8 (5)	V3—O12—V5	114.7 (5)
O2—W1—O4	87.1 (5)	W3—O12—W5	114.7 (5)
O5—W1—O4	87.0 (4)	W3—O13—W6	115.8 (6)
O1—W1—O3	102.5 (5)	W5—O15—W4	115.4 (5)
O2—W1—O3	88.2 (5)	W6—O16—W4	116.3 (6)
O5—W1—O3	86.2 (4)	W5—O18—W6	116.4 (5)
O4—W1—O3	153.7 (4)	O20—W7—O23	103.2 (5)
O1—W1—O6	179.3 (5)	O20—W7—O22	102.8 (5)
O2—W1—O6	78.0 (4)	O23—W7—O22	89.7 (5)
O5—W1—O6	76.3 (4)	O20—W7—O24	103.9 (5)
O4—W1—O6	76.8 (4)	O23—W7—O24	86.7 (4)
O3—W1—O6	76.9 (4)	O22—W7—O24	153.2 (4)
O1—W1—W2	134.1 (4)	O20—W7—O21	103.8 (5)
O2—W1—W2	32.4 (3)	O23—W7—O21	152.9 (4)
O5—W1—W2	122.0 (3)	O22—W7—O21	86.5 (4)
O4—W1—W2	80.2 (3)	O24—W7—O21	84.8 (4)
O3—W1—W2	82.0 (3)	O20—W7—O25	179.7 (4)
O6—W1—W2	45.7 (3)	O23—W7—O25	77.0 (3)
O7—W2—O2	103.2 (5)	O22—W7—O25	77.0 (3)
O7—W2—O9	104.9 (5)	O24—W7—O25	76.3 (3)
O2—W2—O9	88.0 (5)	O21—W7—O25	76.0 (3)
O7—W2—O8	100.8 (5)	O26—W8—O21	101.9 (5)
O2—W2—O8	86.9 (5)	O26—W8—O27	101.0 (5)
O9—W2—O8	154.3 (5)	O21—W8—O27	88.4 (5)
O7—W2—O10	101.8 (5)	O26—W8—O23^{i}	103.9 (5)
O2—W2—O10	154.8 (5)	O21—W8—O23^{i}	154.2 (5)
O9—W2—O10	88.3 (5)	O27—W8—O23^{i}	86.7 (5)
O8—W2—O10	85.7 (5)	O26—W8—O28^{i}	103.7 (5)
O7—W2—O6	177.8 (5)	O21—W8—O28^{i}	87.1 (5)
O2—W2—O6	77.7 (4)	O27—W8—O28^{i}	155.3 (5)
O9—W2—O6	77.1 (4)	O23^{i}—W8—O28^{i}	86.8 (4)
O8—W2—O6	77.1 (4)	O26—W8—O25	178.8 (4)
O10—W2—O6	77.2 (4)	O21—W8—O25	77.4 (3)
O7—W2—W1	135.6 (4)	O27—W8—O25	78.1 (3)
O2—W2—W1	32.4 (3)	O23^{i}—W8—O25	76.8 (3)

O9—W2—W1	81.0 (3)	O28^{i}—W8—O25	77.2 (3)
O8—W2—W1	81.2 (3)	O26—W8—W9^{i}	135.6 (4)
O10—W2—W1	122.5 (3)	O21—W8—W9^{i}	80.4 (3)
O6—W2—W1	45.3 (3)	O27—W8—W9^{i}	123.4 (3)
O7—W2—W3	132.5 (4)	O23^{i}—W8—W9^{i}	81.2 (3)
O2—W2—W3	81.3 (3)	O28^{i}—W8—W9^{i}	31.9 (3)
O9—W2—W3	122.6 (3)	O25—W8—W9^{i}	45.27 (2)
O8—W2—W3	31.7 (3)	O29—W9—O28	102.8 (5)
O10—W2—W3	79.8 (3)	O29—W9—O24^{i}	101.5 (5)
O6—W2—W3	45.4 (3)	O28—W9—O24^{i}	88.6 (4)
W1—W2—W3	60.74 (3)	O29—W9—O22	104.0 (5)
O11—W3—O8	105.8 (5)	O28—W9—O22	88.0 (5)
O11—W3—O13	104.4 (5)	O24^{i}—W9—O22	154.4 (5)
O8—W3—O13	90.2 (5)	O29—W9—O27	102.8 (5)
O11—W3—O3	100.2 (5)	O28—W9—O27	154.4 (4)
O8—W3—O3	88.9 (5)	O24^{i}—W9—O27	86.7 (4)
O13—W3—O3	154.7 (5)	O22—W9—O27	85.5 (5)
O11—W3—O12	99.6 (5)	O29—W9—O25	179.0 (4)
O8—W3—O12	154.5 (5)	O28—W9—O25	77.6 (3)
O13—W3—O12	85.5 (5)	O24^{i}—W9—O25	77.5 (3)
O3—W3—O12	84.6 (5)	O22—W9—O25	77.0 (3)
O11—W3—O6	175.1 (5)	O27—W9—O25	76.8 (3)
O8—W3—O6	78.4 (4)	O29—W9—W8^{i}	135.1 (4)
O13—W3—O6	77.9 (4)	O28—W9—W8^{i}	32.3 (3)
O3—W3—O6	77.2 (4)	O24^{i}—W9—W8^{i}	81.5 (3)
O12—W3—O6	76.1 (4)	O22—W9—W8^{i}	82.1 (3)
O11—W3—W2	138.6 (4)	O27—W9—W8^{i}	122.1 (3)
O8—W3—W2	32.8 (3)	O25—W9—W8^{i}	45.29 (2)
O13—W3—W2	83.3 (3)	O20—V7—O23	103.2 (5)
O3—W3—W2	82.3 (3)	O20—V7—O22	102.8 (5)
O12—W3—W2	121.7 (3)	O23—V7—O22	89.7 (5)
O6—W3—W2	45.6 (3)	O20—V7—O24	103.9 (5)
O14—W4—O15	104.5 (6)	O23—V7—O24	86.7 (4)
O14—W4—O16	103.6 (5)	O22—V7—O24	153.2 (4)
O15—W4—O16	89.9 (5)	O20—V7—O21	103.8 (5)
O14—W4—O4	103.1 (5)	O23—V7—O21	152.9 (4)

O15—W4—O4	88.5 (5)	O22—V7—O21	86.5 (4)
O16—W4—O4	152.8 (5)	O24—V7—O21	84.8 (4)
O14—W4—O9	101.4 (6)	O20—V7—O25	179.7 (4)
O15—W4—O9	154.0 (5)	O23—V7—O25	77.0 (3)
O16—W4—O9	86.0 (5)	O22—V7—O25	77.0 (3)
O4—W4—O9	83.7 (4)	O24—V7—O25	76.3 (3)
O14—W4—O6	177.6 (5)	O21—V7—O25	76.0 (3)
O15—W4—O6	77.8 (4)	O26—V8—O21	101.9 (5)
O16—W4—O6	77.0 (4)	O26—V8—O27	101.0 (5)
O4—W4—O6	76.1 (4)	O21—V8—O27	88.4 (5)
O9—W4—O6	76.2 (4)	O26—V8—O23^{i}	103.9 (5)
O14—W4—W5	136.8 (4)	O21—V8—O23^{i}	154.2 (5)
O15—W4—W5	32.3 (3)	O27—V8—O23^{i}	86.7 (5)
O16—W4—W5	82.2 (3)	O26—V8—O28^{i}	103.7 (5)
O4—W4—W5	82.0 (3)	O21—V8—O28^{i}	87.1 (5)
O9—W4—W5	121.8 (3)	O27—V8—O28^{i}	155.3 (5)
O6—W4—W5	45.6 (3)	O23^{i}—V8—O28^{i}	86.8 (4)
O17—W5—O15	103.5 (5)	O26—V8—O25	178.8 (4)
O17—W5—O18	102.3 (5)	O21—V8—O25	77.4 (3)
O15—W5—O18	88.6 (5)	O27—V8—O25	78.1 (3)
O17—W5—O5	103.4 (5)	O23^{i}—V8—O25	76.8 (3)
O15—W5—O5	88.0 (5)	O28^{i}—V8—O25	77.2 (3)
O18—W5—O5	154.2 (5)	O29—V9—O28	102.8 (5)
O17—W5—O12	101.0 (5)	O29—V9—O24^{i}	101.5 (5)
O15—W5—O12	155.6 (5)	O28—V9—O24^{i}	88.6 (4)
O18—W5—O12	86.5 (5)	O29—V9—O22	104.0 (5)
O5—W5—O12	86.1 (5)	O28—V9—O22	88.0 (5)
O17—W5—O6	177.5 (5)	O24^{i}—V9—O22	154.4 (5)
O15—W5—O6	79.0 (4)	O29—V9—O27	102.8 (5)
O18—W5—O6	77.4 (4)	O28—V9—O27	154.4 (4)
O5—W5—O6	76.8 (4)	O24^{i}—V9—O27	86.7 (4)
O12—W5—O6	76.6 (4)	O22—V9—O27	85.5 (5)
O17—W5—W4	135.8 (4)	O29—V9—O25	179.0 (4)
O15—W5—W4	32.3 (3)	O28—V9—O25	77.6 (3)
O18—W5—W4	81.4 (3)	O24^{i}—V9—O25	77.5 (3)
O5—W5—W4	82.0 (3)	O22—V9—O25	77.0 (3)

O12—W5—W4	123.2 (3)	O27—V9—O25	76.8 (3)
O6—W5—W4	46.7 (3)	W8—O21—W7	116.0 (5)
O17—W5—W6	134.2 (4)	V8—O21—V7	116.0 (5)
O15—W5—W6	82.4 (4)	V7—O22—V9	116.3 (5)
O18—W5—W6	31.9 (3)	W7—O22—W9	116.3 (5)
O5—W5—W6	122.3 (3)	W7—O23—W8^{i}	116.7 (5)
O12—W5—W6	80.8 (3)	W9^{i}—O24—W7	115.8 (5)
O6—W5—W6	45.5 (3)	W9^{i}—O25—W9	180.0
W4—W5—W6	60.79 (3)	W9^{i}—O25—W8	89.44 (3)
O19—W6—O18	103.7 (5)	W9—O25—W8	90.56 (3)
O19—W6—O16	104.1 (5)	V9—O25—V8	90.56 (3)
O18—W6—O16	87.7 (5)	W9^{i}—O25—W8^{i}	90.56 (3)
O19—W6—O10	102.6 (5)	W9—O25—W8^{i}	89.44 (3)
O18—W6—O10	153.7 (5)	W8—O25—W8^{i}	180.0
O16—W6—O10	87.2 (5)	W9^{i}—O25—W7	90.39 (3)
O19—W6—O13	101.8 (5)	W9—O25—W7	89.60 (3)
O18—W6—O13	86.8 (4)	W8—O25—W7	90.59 (4)
O16—W6—O13	154.1 (5)	W8^{i}—O25—W7	89.41 (4)
O10—W6—O13	86.6 (5)	V9—O25—V7	89.60 (3)
O19—W6—O6	178.5 (5)	V8—O25—V7	90.59 (4)
O18—W6—O6	76.5 (4)	W9^{i}—O25—W7^{i}	89.60 (3)
O16—W6—O6	77.4 (4)	W9—O25—W7^{i}	90.40 (4)
O10—W6—O6	77.2 (4)	W8—O25—W7^{i}	89.41 (4)
O13—W6—O6	76.7 (4)	W8^{i}—O25—W7^{i}	90.59 (4)
O19—W6—W5	135.3 (4)	W7—O25—W7^{i}	180.0
O18—W6—W5	31.6 (3)	V8—O27—V9	114.5 (5)
O16—W6—W5	81.0 (3)	W8—O27—W9	114.5 (5)
O10—W6—W5	122.1 (3)	W9—O28—W8^{i}	115.8 (5)
O13—W6—W5	81.1 (3)	H30A—O30—H30B	110.6 (19)
O6—W6—W5	44.8 (3)	H31A—O31—H31B	152.050 (6)
O1—V1—O2	101.8 (5)	H32A—O32—H32B	114 (2)
O1—V1—O5	103.9 (5)	H33A—O33—H33B	110.5 (18)
O2—V1—O5	154.3 (5)	H34A—O34—H34B	95 (10)
O1—V1—O4	103.8 (5)	H35A—O35A—H35B	110.4 (19)
O2—V1—O4	87.1 (5)	H35C—O35B—H35D	110.5 (18)
O5—V1—O4	87.0 (4)	C1—N1—H1A	88 (10)

O1—V1—O3	102.5 (5)	C1—N1—H1B	107 (9)
O2—V1—O3	88.2 (5)	H1A—N1—H1B	111.0 (19)
O5—V1—O3	86.2 (4)	C1—N1—H1C	126 (10)
O4—V1—O3	153.7 (4)	H1A—N1—H1C	111.0 (19)
O1—V1—O6	179.3 (5)	H1B—N1—H1C	111.0 (19)
O2—V1—O6	78.0 (4)	C2—N2—C3	113.8 (13)
O5—V1—O6	76.3 (4)	C2—N2—H2A	103 (10)
O4—V1—O6	76.8 (4)	C3—N2—H2A	126 (10)
O3—V1—O6	76.9 (4)	C2—N2—H2B	96 (10)
O7—V2—O2	103.2 (5)	C3—N2—H2B	100 (10)
O7—V2—O9	104.9 (5)	H2A—N2—H2B	114 (10)
O2—V2—O9	88.0 (5)	C4—N3—H3A	109.5
O7—V2—O8	100.8 (5)	C4—N3—H3B	109.5
O2—V2—O8	86.9 (5)	H3A—N3—H3B	109.5
O9—V2—O8	154.3 (5)	C4—N3—H3C	109.5
O7—V2—O10	101.8 (5)	H3A—N3—H3C	109.5
O2—V2—O10	154.8 (5)	H3B—N3—H3C	109.5
O9—V2—O10	88.3 (5)	N1—C1—C2	115.5 (15)
O8—V2—O10	85.7 (5)	N1—C1—H1D	108.4
O7—V2—O6	177.8 (5)	C2—C1—H1D	108.4
O2—V2—O6	77.7 (4)	N1—C1—H1E	108.4
O9—V2—O6	77.1 (4)	C2—C1—H1E	108.4
O8—V2—O6	77.1 (4)	H1D—C1—H1E	107.5
O10—V2—O6	77.2 (4)	C1—C2—N2	112.7 (14)
O11—V3—O8	105.8 (5)	C1—C2—H2C	109.1
O11—V3—O13	104.4 (5)	N2—C2—H2C	109.1
O8—V3—O13	90.2 (5)	C1—C2—H2D	109.1
O11—V3—O3	100.2 (5)	N2—C2—H2D	109.1
O8—V3—O3	88.9 (5)	H2C—C2—H2D	107.8
O13—V3—O3	154.7 (5)	C4—C3—N2	113.2 (15)
O11—V3—O12	99.6 (5)	C4—C3—H3D	108.9
O8—V3—O12	154.5 (5)	N2—C3—H3D	108.9
O13—V3—O12	85.5 (5)	C4—C3—H3E	108.9
O3—V3—O12	84.6 (5)	N2—C3—H3E	108.9
O11—V3—O6	175.1 (5)	H3D—C3—H3E	107.7
O8—V3—O6	78.4 (4)	C3—C4—N3	115.9 (16)

O13—V3—O6	77.9 (4)	C3—C4—H4A	108.3
O3—V3—O6	77.2 (4)	N3—C4—H4A	108.3
O12—V3—O6	76.1 (4)	C3—C4—H4B	108.3
O17—V5—O15	103.5 (5)	N3—C4—H4B	108.3
O17—V5—O18	102.3 (5)	H4A—C4—H4B	107.4
O15—V5—O18	88.6 (5)	C5—N4—H4C	109.5
O17—V5—O5	103.4 (5)	C5—N4—H4D	109.5
O15—V5—O5	88.0 (5)	H4C—N4—H4D	109.5
O18—V5—O5	154.2 (5)	C5—N4—H4E	109.5
O17—V5—O12	101.0 (5)	H4C—N4—H4E	109.5
O15—V5—O12	155.6 (5)	H4D—N4—H4E	109.5
O18—V5—O12	86.5 (5)	C6—N5—C7	114.2 (15)
O5—V5—O12	86.1 (5)	C6—N5—H5A	131 (10)
O17—V5—O6	177.5 (5)	C7—N5—H5A	93 (10)
O15—V5—O6	79.0 (4)	C6—N5—H5B	102 (10)
O18—V5—O6	77.4 (4)	C7—N5—H5B	103 (10)
O5—V5—O6	76.8 (4)	H5A—N5—H5B	111 (10)
O12—V5—O6	76.6 (4)	C8—N6—H6A	109.5
W2—O2—W1	115.3 (5)	C8—N6—H6B	109.5
V2—O2—V1	115.3 (5)	H6A—N6—H6B	109.5
W3—O3—W1	115.3 (5)	C8—N6—H6C	109.5
V3—O3—V1	115.3 (5)	H6A—N6—H6C	109.5
W4—O4—W1	116.3 (5)	H6B—N6—H6C	109.5
W5—O5—W1	116.1 (5)	N4—C5—C6	108.5 (14)
V5—O5—V1	116.1 (5)	N4—C5—H5C	110.0
W5—O6—W1	90.9 (4)	C6—C5—H5C	110.0
V5—O6—V1	90.9 (4)	N4—C5—H5D	110.0
W5—O6—W3	92.7 (4)	C6—C5—H5D	110.0
W1—O6—W3	90.5 (4)	H5C—C5—H5D	108.4
V5—O6—V3	92.7 (4)	N5—C6—C5	111.5 (15)
V1—O6—V3	90.5 (4)	N5—C6—H6D	109.3
W5—O6—W2	178.4 (5)	C5—C6—H6D	109.3
W1—O6—W2	89.0 (4)	N5—C6—H6E	109.3
W3—O6—W2	89.0 (4)	C5—C6—H6E	109.3
V5—O6—V2	178.4 (5)	H6D—C6—H6E	108.0
V1—O6—V2	89.0 (4)	C8—C7—N5	112.3 (14)

V3—O6—V2	89.0 (4)	C8—C7—H7A	109.2
W5—O6—W6	89.6 (4)	N5—C7—H7A	109.2
W1—O6—W6	179.5 (6)	C8—C7—H7B	109.2
W3—O6—W6	89.6 (4)	N5—C7—H7B	109.2
W2—O6—W6	90.5 (4)	H7A—C7—H7B	107.9
W5—O6—W4	87.8 (4)	N6—C8—C7	111.9 (14)
W1—O6—W4	90.7 (4)	N6—C8—H8A	109.2
W3—O6—W4	178.7 (5)	C7—C8—H8A	109.2
W2—O6—W4	90.6 (4)	N6—C8—H8B	109.2
W6—O6—W4	89.1 (4)	C7—C8—H8B	109.2
W3—O8—W2	115.5 (6)	H8A—C8—H8B	107.9

Table S3: Hydrogen bonds (in Å) for $(C_2H_8N)_6[V_2Mo_{18}O_{62}].3H_2O$.

**D-H**	**d(D-H)**	**d(H..A)**	**<DHA**	**d(D..A)**	**A**	
O63-H47	0.993	1.852	150.00	2.757	O24	
O63-H48	1.215	2.493	136.34	3.475	O66	
O63-H48	1.215	2.462	164.79	3.649	O66	[-x+1, -y+1, -z+1]
O66-H1	1.232	2.584	118.40	3.350	O40	[-x+1, -y+1, -z+1]
O66-H2	1.268	2.379	140.51	3.453	O44	[x+1/2, -y+1/2, z+1/2]
N1-H11	0.970	1.923	152.78	2.821	O13	[-x+1/2, y+1/2, -z+1/2]
N1-H11	0.970	2.549	128.14	3.239	O33	[-x+1/2, y+1/2, -z+1/2]
N1-H11	0.970	2.430	116.13	2.987	O54	[x, y+1, z]
N1-H49	0.970	2.019	152.93	2.917	O7	
N1-H49	0.970	2.626	132.16	3.355	O31	
C1-H6	0.960	2.566	172.89	3.521	O58	[x, y+1, z]
C1-H5	0.960	2.552	149.65	3.415	O62	
C1-H7	0.960	2.653	142.24	3.462	O44	[-x+1/2, y+1/2, -z+1/2]
C2-H8	0.960	2.514	125.27	3.167	O15	[x, y+1, z]
C2-H9	0.960	2.603	131.82	3.321	O33	[-x+1/2, y+1/2, -z+1/2]
C2-H10	0.960	2.624	136.06	3.381	O37	
C2-H10	0.960	2.584	131.24	3.297	O47	
C2-H10	0.960	2.507	155.64	3.405	O52	
N2-H18	0.970	1.928	156.60	2.845	O61	[-x+3/2, y+1/2, -z+1/2]
N2-H50	0.970	2.253	135.63	3.024	O20	
N2-H50	0.970	2.128	148.25	2.997	O37	

C3-H12	0.960	2.636	142.58	3.448	O38	[x, y+1, z]
C4-H15	0.960	2.653	120.08	3.243	O15	[x, y+1, z]
N3-H51	0.970	2.459	138.14	3.247	O64	
N3-H21	0.970	2.006	165.03	2.954	O28	
N3-H21	0.970	2.569	126.64	3.242	O59	
C6-H19	0.960	2.460	155.40	3.357	O63	[-x+1/2, y-1/2, -z+1/2]
C6-H20	0.960	2.600	121.98	3.214	O28	
C6-H22	0.960	2.484	135.03	3.235	O64	
N4-H52	0.970	2.259	125.81	2.934	O38	[x-1/2, -y+1/2, z-1/2]
N4-H52	0.970	1.978	147.90	2.847	O43	[-x+1, -y+1, -z]
C7-H32	0.960	2.636	163.60	3.568	O52	
C7-H30	0.960	2.450	144.46	3.279	O46	[-x+1, -y+1, -z]
C8-H29	0.960	2.444	151.77	3.321	O21	[x-1/2, -y+1/2, z-1/2]
N5-H53	0.970	2.313	138.53	3.107	O22	
N5-H53	0.970	2.307	138.87	3.104	O24	
N5-H39	0.970	1.920	162.07	2.858	O10	[-x+1, -y, -z+1]
N5-H39	0.970	2.567	120.73	3.174	O22	[-x+1, -y, -z+1]
C9-H34	0.960	2.584	169.91	3.533	O21	
C9-H35	0.960	2.487	138.83	3.271	O33	[-x+1, -y, -z+1]
C9-H35	0.960	2.596	136.12	3.354	O56	[x+1/2, -y+1/2, z+1/2]
C10-H37	0.960	2.577	129.32	3.271	O49	[-x+1, -y, -z+1]
C10-H36	0.960	2.545	160.84	3.466	O16	
C10-H36	0.960	2.612	135.53	3.365	O34	
N6-H40	0.970	2.515	118.72	3.100	O46	
N6-H54	0.970	2.489	114.40	3.021	O19	[-x+1, -y, -z]
N6-H54	0.970	1.862	162.53	2.802	O45	[x-1/2, -y+1/2, z-1/2]
C11-H41	0.960	2.637	114.56	3.159	O19	[-x+1, -y, -z]
C12-H46	0.960	2.594	115.06	3.124	O46	
C12-H44	0.960	2.637	119.65	3.221	O15	[-x+1, -y, -z]
C12-H44	0.960	2.614	122.66	3.235	O19	[-x+1, -y, -z]

Table S4: Hydrogen bonds (in Å) for $(C_4H_{16}N_3)_4[V_2W_4O_{19}]_3.12H_2O$

D*—H⋯*A	***D*—H**	**H⋯*A***	***D*⋯*A***	***D*—H⋯*A***
O30—H30*B*⋯O9[ii]	0.84 (1)	2.25 (7)	3.008 (15)	150 (12)

O30—H30*B*···N4a^{iii}	0.84 (1)	2.64 (8)	3.33 (2)	140 (11)
O31—H31*B*···O8^{iv}	0.76 (1)	2.45 (1)	2.845 (11)	114 (1)
O32—H32*B*···O27^{i}	0.85 (1)	2.15 (14)	2.836 (17)	138 (18)
O33—H33*A*···O24^{v}	0.87 (1)	2.09 (10)	2.862 (16)	147 (16)
O33—H33*B*···O19^{vi}	0.87 (1)	2.65 (10)	3.390 (19)	144 (15)
O33—H33*B*···O26^{vii}	0.87 (1)	2.34 (14)	2.939 (18)	127 (14)
O34—H34*A*···O18^{iv}	0.95 (2)	2.11 (7)	3.04 (2)	165 (22)
O35*A*a—H35*A*a···O2^{viii}	0.87 (1)	2.15 (18)	2.81 (2)	132 (20)
O35*B*b—H35*C*b···O4	0.87 (1)	2.2 (2)	2.85 (6)	130 (27)
O35*B*b—H35*D*b···O31^{ix}	0.87 (1)	2.6 (3)	3.39 (6)	150 (47)
N1a—H1*A*a···O12	0.85 (1)	2.22 (5)	3.041 (18)	161 (11)
N1a—H1*A*a···N2a	0.85 (1)	2.64 (11)	3.21 (2)	126 (11)
N1a—H1*B*a···O31	0.85 (1)	2.09 (6)	2.859 (15)	150 (11)
N1a—H1*C*a···O5	0.85 (1)	2.66 (12)	3.076 (18)	112 (10)
N1a—H1*C*a···O10^{iv}	0.85 (1)	2.01 (5)	2.824 (19)	160 (13)
N2^{a}—H2*A*a···O32^{v}	0.82 (1)	1.94 (9)	2.68 (2)	149 (17)
N2a—H2*B*a···O12	0.82 (1)	1.87 (7)	2.638 (18)	155 (16)
N3a—H3*A*a···O21^{x}	0.89	2.12	2.963 (17)	159
N3a—H3*B*a···O33^{vi}	0.89	2.00	2.88 (2)	174
N3a—H3*C*a···O12	0.89	2.47	3.205 (18)	140
N3a—H3*C*a···O13	0.89	2.20	2.998 (18)	150
C1a—H1*D*a···O13^{iv}	0.97	2.65	3.32 (2)	127
C1a—H1*D*a···O29^{xi}	0.97	2.64	3.35 (2)	130
C1a—H1*E*a···O22^{xi}	0.97	2.63	3.15 (2)	114
C2a—H2*D*a···O29^{xi}	0.97	2.60	3.33 (2)	132
C3a—H3*E*a···O26^{x}	0.97	2.44	3.39 (2)	167
C4a—H4*A*a···O28^{xii}	0.97	2.59	3.19 (2)	120
C4a—H4*A*a···O33	0.97	2.61	3.09 (2)	111
C4a—H4*B*a···O29^{xiii}	0.97	2.41	3.16 (2)	134
N4a—H4*C*a···O14^{ii}	0.89	2.48	3.054 (18)	123
N4a—H4*C*a···O31^{viii}	0.89	2.54	3.177 (15)	129
N4a—H4*D*a···O9^{iv}	0.89	1.92	2.787 (17)	163

N4a—H4*E*a···O1^{viii}	0.89	2.18	2.969 (18)	148
N4a—H4*E*a···O7^{xiv}	0.89	2.40	3.059 (19)	131
N5a—H5*A*a···O35*A*a	0.82 (1)	1.96 (10)	2.69 (2)	148 (19)
N5a—H5*B*a···O30	0.82 (1)	1.98 (7)	2.76 (2)	158 (19)
N6a—H6*A*a···O17^{iii}	0.89	2.06	2.908 (19)	159
N6a—H6*B*a···O20^{iii}	0.89	2.26	3.137 (19)	167
N6a—H6*C*a···O11^{viii}	0.89	2.37	3.08 (2)	137
N6a—H6*C*a···O27^{xv}	0.89	2.48	3.090 (18)	126
C5a—H5*D*a···O14^{ii}	0.97	2.66	3.15 (2)	112
C6a—H6*D*a···O3^{viii}	0.97	2.36	3.27 (2)	156
C6a—H6*E*a···O31^{viii}	0.97	2.61	3.177 (18)	118
C7a—H7*A*a···O15^{iii}	0.97	2.54	3.46 (2)	158
C7a—H7*B*a···O11^{viii}	0.97	2.53	3.26 (2)	132
C8a—H8*B*a···O16^{ii}	0.97	2.46	3.39 (2)	162
C8a—H8*B*a···O19^{ii}	0.97	2.51	3.18 (2)	126

Symmetry codes: (i) −*x*, −*y*+1, −*z*+2; (ii) *x*−1, −*y*+1/2, *z*−1/2; (iii) *x*, −*y*+1/2, *z*−1/2; (iv) *x*−1, *y*, *z*; (v) −*x*+1, −*y*+1, −*z*+1; (vi) −*x*+2, −*y*+1, −*z*+1; (vii) −*x*, −*y*+1, −*z*+1; (viii) *x*, −*y*+1/2, *z*+1/2; (ix) *x*+1, −*y*+1/2, *z*+1/2; (x) *x*+1, *y*, *z*; (xi) *x*, *y*, *z*−1; (xii) −*x*+1, −*y*+1, −*z*+2; (xiii) *x*+1, *y*, *z*−1; (xiv) *x*−1, −*y*+1/2, *z*+1/2; (xv) *x*+1, −*y*+1/2, *z*−1/2.

Table S5. Crystal data and structure refinement parameters for compounds 1 and 2.

**Empirical formula**	**$(C_2H_8N_1)_6[V_2Mo_{18}O_{62}].3H_2O$ (1)**	**$(C_4H_{16}N_3)_4[V_2W_4O_{19}]_3.12H_2O$ (2)**
Formula weight (g.mol $^{-1}$)	3150.98	4064.82
Temperature (K)	109	296
Crystal system	Monoclinic	Monoclinic
Space group	$P2_1/n$	$P2_1/c$
a (˚ A)	23.1645 (11)	8.5140 (16)
b (˚ A)	13.5966	43.351 (9)
c (˚ A)	24.5135 (11)	10.791 (2)
β (°)	115.926 (2)	107.264 (5)
V (˚A ^{3})	6943.7 (6)	3803.5 (13)
Z space group	2	2
μ (mm−1)	3.50	18.88
F(000)	5718	3692
Index ranges	-30 < h < 30, -18 < k < 18, -32 < l < 32	-10< h < 10, -54 < k < 54, -13 < l < 13
Radiation (˚ A)	MoKα (λ= 0.71073 Å)	MoKα (λ= 0.71073 Å)
Independent reflections;	17192	7489
R_{int}	0.027	0.129
Reflections with I > 2 σ (I)	16540	5270
Refined parameters	911	545
R1(reflections)	0.0406(16540)	0.0637(5270)
wR2(reflections)	0.0945(17192)	0.1094(7489)
S (goodness-of-fit)	1.07	1.06
Δϱmax / Δϱmin	4.05 e Å−3/ −2.94 e Å−3	1.88 e Å−3/−1.92 e Å
